# Optimization of Kerosene-like Fuels Produced via Catalytic Pyrolysis of Packaging Plastic Waste via Central Composite Design and Response Surface Methodology: Performance of Iron-Doped Dolomite and Activated Carbon

**DOI:** 10.3390/molecules30132884

**Published:** 2025-07-07

**Authors:** Oratepin Arjharnwong, Tharapong Vitidsant, Aminta Permpoonwiwat, Naphat Phowan, Witchakorn Charusiri

**Affiliations:** 1 Master of Programme in Environmental Technology and Resources Management, Faculty of Environmental Culture and Ecotourism, Srinakharinwirot University, Bangkok 10110, Thailand; 2 Department of Chemical Technology, Faculty of Science, Chulalongkorn University, Bangkok 10110, Thailand; 3 Centre of Fuels Research and Energy from Biomass, Chulalongkorn University, Saraburi 18110, Thailand; 4 School of Arts and Science, University of Vanderbilt, Nashville, TN 37240, USA; 5 Department of Environment and Resources, Faculty of Environmental Culture and Ecotourism, Srinakharinwirot University, Bangkok 10110, Thailand

**Keywords:** packaging plastic waste, response surface methodology, kerosene-like

## Abstract

Rapid economic growth has led to an increase in the use of multilayer plastic packaging, which involves complex polymer compositions and hinders recycling. This study investigated the catalytic pyrolysis of plastic packaging waste in a 3000 cm^3^ semibatch reactor, aiming to optimize kerosene-like hydrocarbon production. The temperature (420–500 °C), N_2_ flow rate (25–125 mL/min), and catalyst loading (5–20 wt.%) were examined individually and in combination with activated carbon and an Fe-doped dolomite (Fe/DM) catalyst. Central composite design (CCD) and response surface methodology (RSM) were used to identify the optimal conditions and synergistic effects. Pyrolysis product analysis involved simulation distillation gas chromatography (Sim-DGC), gas chromatography/mass spectrometry (GC/MS), and Fourier transform infrared (FT-IR) spectroscopy. The optimal conditions (440 °C, 50 mL/min N_2_ flow, catalyst loading of 10 wt.% using a 5 wt.% Fe-doped dolomite-activated carbon 0.6:0.4 mass/molar ratio) yielded the highest pyrolysis oil (79.6 ± 0.35 wt.%) and kerosene-like fraction (22.3 ± 0.22 wt.%). The positive synergistic effect of Fe/DM and activated carbon (0.6:0.4) enhanced the catalytic activity, promoting long-chain polymer degradation into mid-range hydrocarbons, with secondary cracking yielding smaller hydrocarbons. The pore structure and acid sites of the catalyst improved the conversion of intermediate hydrocarbons into aliphatic compounds (C_5_–C_15_), increasing kerosene-like hydrocarbon production.

## 1. Introduction

Global plastic production and consumption have resulted in a surge in plastic waste; approximately 78% of plastic waste is dumped in landfills or is illegally dumped, while only a small fraction is incinerated (8–12%) or recycled (7–9%) [1]. Plastic packaging is one contributor to this problem driven by global consumerism and societal development [2,3].

The complex composition of packaging materials is a significant barrier to recycling. These plastics, typically based on polyethylene (PE) or polypropylene (PP), are often fused with other materials like adhesives or aluminum foil to improve strength and barrier properties against moisture [2,4]. The presence of various polymers, plasticizers, and additives make them incompatible with conventional recycling streams. Therefore, the unrecyclable plastic packaging waste (PPW) persists in landfills for decades, leaching toxic substances into the soil and water, and exacerbating environmental pollution. While incineration is an alternative, it requires careful management to avoid further environmental harm [5], underscoring the dire need for alternative waste management strategies. One of the most promising solutions lies in the use of advanced thermochemical technologies to convert these unrecyclable plastics into valuable byproducts, thus offering a pathway to mitigate the environmental burden [6,7,8].

Pyrolysis, a thermochemical process that decomposes hydrocarbon materials in an oxygen-free environment, is a viable method for converting nonrecyclable plastic waste into pyrolysis oil. This oil represents a promising alternative fuel that can be refined into valuable hydrocarbons and chemicals [7,9,10]. A significant application is the production of kerosene or jet fuel fractions, which can help meet the aviation industry’ rising demand for sustainable alternatives to fossil resources. This strategy supports a circular economy by converting otherwise non-recyclable plastic waste into high-value energy products, thereby reducing the volume of waste sent to landfills [11]. However, several studies have been conducted on the pyrolysis and copyrolysis of polymers, such as rubber tires, lubricant oil, which have relatively low yields of valuable fuels [12,13,14]. The effectiveness of the process is highly dependent on key factors, including the type of pyrolyzer, operating conditions, and the use of active catalysts.

Dolomite (DM) has gained attention as a basic catalyst for hydrocarbon conversion because of its bond scission and isomerization capabilities [15,16]. It is also readily available and inexpensive for regeneration, thus increasing the catalytic activity for further use. Increasing the acid strength to promote the catalytic activity of dolomite can be further improved by incorporating a metal oxide to improve its acid strength and increase the number of active sites. Therefore, the incorporation of iron oxide into dolomite also plays a significant role in both the acidic and basic characteristics of the catalyst, promoting the catalytic conversion of larger hydrocarbons into smaller, more valuable compounds and enhancing the isomerization and cracking processes that are essential for upgrading pyrolysis oils into high-quality fuels [17,18,19,20], such as kerosene-like fractions. Similarly, activated carbon (AC) is another catalyst support with a high surface area and textural properties that is commonly used for various catalytic reactions [21]. When combined with metal impregnation into dolomite, the surface of activated carbon can be further modified to improve its catalytic performance, facilitating carbon-carbon bond scission into small hydrocarbon compounds and allowing better accessibility and selective hydrocarbon transformations, thus promoting the formation of smaller, branched hydrocarbons with desirable fuel properties [9,10,12,18,19,22].

A key aspect of this research is optimizing production processes to increase yield, improve efficiency, and reduce operational costs via a systematic design of experiments, which enables the identification of critical factors that influence the determination of optimal operating conditions. The central composite design (CCD) implemented within response surface methodology contributes to the advancement of sustainable experiments [23,24], facilitating the development of a quadratic response model that accurately represents the relationship between operating conditions and the desirable yield of, in particular, kerosene-like fuels from catalytic pyrolysis [25]. Additionally, the synergy between the enhanced properties of each Fe-impregnated dolomite and activated carbon catalyst was investigated. The combined increase in acid strength, active site density, and pore structure led to an overall improvement in catalytic pyrolysis, resulting in more efficient cracking and isomerization to produce smaller hydrocarbons with properties similar to those of kerosene [9,18,21,26,27], particularly for the pyrolysis of PPW. The dual catalysts represent a promising route to reduce plastic waste accumulation while contributing to the conversion of hydrocarbon compounds. This work seeks to provide insight into ways to improve suitable aviation practices and address both waste management and energy requirements simultaneously.

## 2. Results and Discussions

### 2.1. Characterization of Feedstock

**Figure 1:**
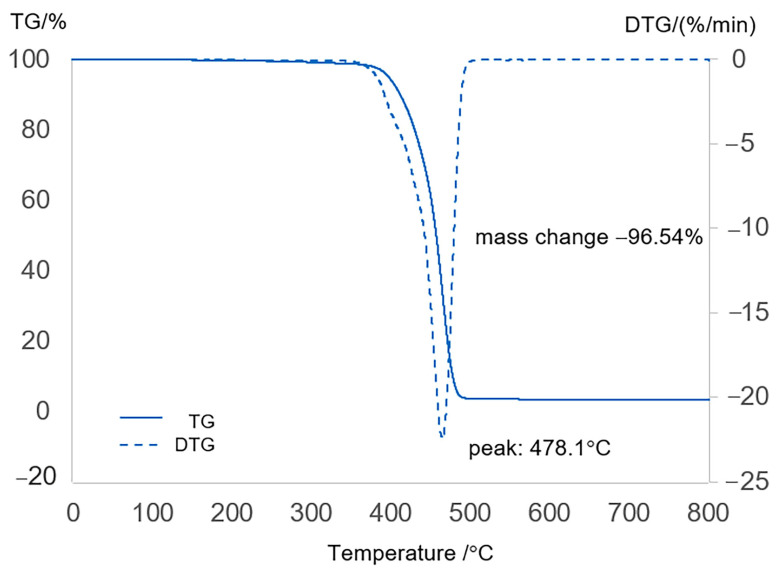
Thermogravimetric analysis curves for PPW.

Thermogravimetric analysis (TGA) of the packaging plastic waste compared with polyethylene (PE) and polypropylene (PP) waste was carried out with a Pyris Diamond TGA/DTG instrument (PerkinElmer, Shelton, WA, USA), which measures the stability of materials by tracking changes in the weight of a sample over a temperature range of 40–800 °C under a nitrogen gas atmosphere with a flow rate of 50 mL/min at a standard temperature scanning rate of 10 °C/min. A sample volume of 50 mg was placed in an aluminum crucible inside an aluminum chamber and heated under an inert atmosphere. The difference in mass of the sample with respect to temperature was measured against time. Figure 1 reveals that the steepest slope, with the highest rates of thermal decomposition and maximum mass loss, occurred within a specific temperature range. This critical temperature range was subsequently used as the baseline parameter to establish a sustainable experimental design and appropriate operating conditions for pyrolysis, ensuring that the temperature was not lower than the identified threshold. Additionally, the thermogravimetric analysis curves of PPW also demonstrated a broad weight loss interval from 30 to 800 °C. The gradual degradation of PPW from 100 °C to 394 °C was attributed primarily to thermal degradation through free radical reactions, resulting in the cleavage of C-H and C-C bonds, followed by increased β-scission of hydrocarbons. A dramatic degradation phase occurred between 410 °C and 505.1 °C; the initial mass loss of the PPW was half of its initial weight at approximately 404.55 °C, and complete degradation occurred at approximately 491.4 °C. Compared with the results of the thermogravimetric analysis of PE and PP (as shown in Figures S1 and S2) over the same temperature range (40–800 °C), distinct weight loss patterns were revealed. PE exhibited a percentage weight change between 435 °C and 500 °C, and the peak thermal degradation of PE occurred at approximately 475.4 °C. While PP showed a weight change between 410 °C and 500 °C, the maximum thermal degradation of PP therefore exhibited a peak at approximately 500 °C. Analysis of the wt.% residual content via TGA revealed that both PE and PP had very low wt.% contents. residualities of 0.29% and 0.14% for PE and PP, respectively. In contrast, the residual weight for PPW was significantly greater at 3.46%, and the difference between the dTG curves of PPW and other plastics indicates the complexity of the PPW compared with that of PE and PP. Therefore, both the residual weights of PE and PP were remarkably similar, whereas the residual weight percentage of PPW was significantly greater because its composite nature, which contains both polymers and nonplastic components that make up PPW, is designed to blend to suit specific application requirements, such as lamination with aluminum foil and adhesives between film layers [1,2]. Therefore, thermogravimetric analysis (TGA) of the thermal properties of plastics provides critical insights into their thermal decomposition behavior at optimal temperatures, which can be applied to determine the temperature ranges for significant weight loss and serve as crucial parameters and operating conditions for the thermal decomposition of different plastic types and their components.

Proximate analysis is a preliminary method used to determine the moisture, volatile matter, ash, and fixed carbon contents of a material, providing an initial indication of its basic composition. This analysis was conducted in accordance with ASTM D7582.

Ultimate analysis, on the other hand, determines the elemental composition, including carbon, hydrogen, nitrogen, and oxygen, and was performed following ASTM D5373. The heating value was measured via a bomb calorimeter in accordance with ASTM D5865. Table 1 presents the results of the proximate analysis, ultimate analysis, and heating value for PPW in comparison with those of PE and PP. For PP, proximate analysis revealed a composition of 97.24 wt.% volatile matter and 2.76 wt.% fixed carbon, with no detectable ash. Ultimate analysis revealed 84.11% carbon, 15.89% hydrogen, and no detectable nitrogen or oxygen. The hydrogen-to-carbon ratio was 2.27, whereas the oxygen-to-carbon ratio was not applicable because of the absence of oxygen. The heating value was 22.31 MJ/kg. For PE, proximate analysis revealed 96.13 wt.% volatile matter, 3.87 wt.% fixed carbon, and no detectable ash. Ultimate analysis revealed 83.24% carbon and 16.76% hydrogen, with no detectable nitrogen or oxygen. The hydrogen-to-carbon ratio was 2.42, and the oxygen-to-carbon ratio was not applicable. The heating value was 23.28 MJ/kg.

For PPW, proximate analysis revealed 92.28 wt.% volatile matter, 2.89 wt.% fixed carbon, and 4.83% ash by weight. Ultimate analysis revealed 83.35% carbon by weight, 14.73% hydrogen, and no detectable nitrogen or oxygen. The hydrogen-to-carbon ratio was 2.12, and the oxygen-to-carbon ratio was 0.02, which were similar to the results of the analyses of the oxygen content in PP. The heating value was 20.45 MJ/kg. Additionally, the heating value of PPW was lower than that of PE and PP. This difference is likely because plastic packaging often comprises a mixture of PP and PE, along with binding components such as adhesives or other polymer materials. These additives increase the thickness, strength, and durability of plastic packaging.

### 2.2. Characterization of the Catalysts

**Figure 2:**
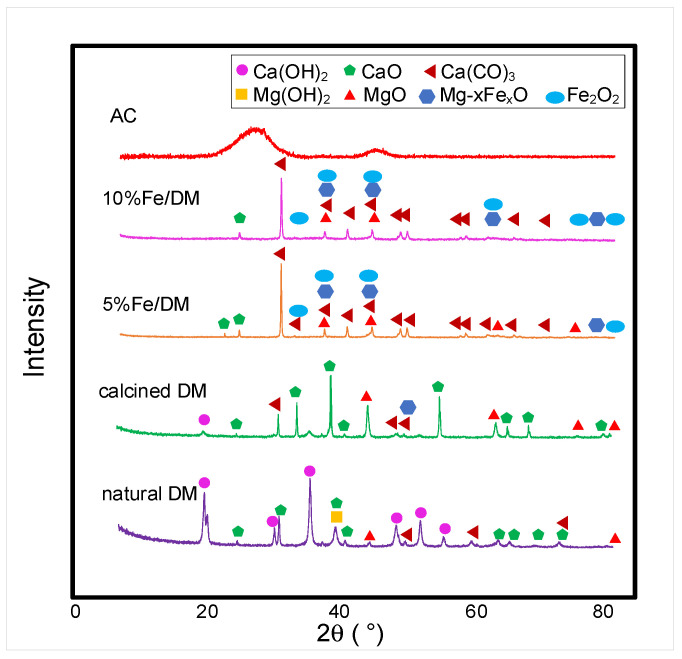
XRD pattern of the catalyst used.

X-ray diffraction (XRD) was performed with an X-ray diffractometer to analyze the catalyst. Figure 2 shows the X-ray diffraction patterns of the catalysts examined in this study. For activated carbon, the XRD analysis revealed no sharp peaks; instead, two broad diffraction peaks appeared at 2θ values ranging from 20° to 30° and 40° to 45°.

The calcined dolomite (DM) primarily consists of CaCO_3_, Ca(OH)_2_, and MgO, which also lack the diffractogram of the Mg(OH)_2_ peak due to thermal decomposition during calcination. Instead, the XRD diffractograms present peaks corresponding to the crystallinity of CaO and MgO [16].

With respect to the modification of dolomite by wet impregnation with Fe(NO_3_)_3_·9H_2_O, the diffraction pattern indicated that the primary structure remained largely unchanged compared with that of calcined dolomite, which consists primarily of CaCO_3_ and MgO components. Figure 2 also shows the XRD patterns of the DM impregnated with various weight percentages of ferric oxide compared with the XRD pattern of the DM. Additional peaks corresponding to Mg_x_Fe_x_O were observed at 2θ values of 36.5°, 42.5°, 61.4°, 73.4°, and 77.6°, along with peaks associated with Fe_2_O_3_ (JCPDS No. 39-1346) at 23.5°, 31.4°, 36.5°, 42.5°, 61.2°, 74.4°, and 78.8°. The presence of these peaks confirms the successful incorporation of ferric oxide into the dolomite support structure [26,27]. The diffraction peaks also suggest that the incorporation of ferric oxide into the dolomite template does not significantly alter the support structure. Notably, the intensity of the peaks representing the calcined dolomite structure decreased following ferric oxide impregnation. The modification of the Fe-doped dolomite template from 5 wt.% to 10 wt.% resulted in the deposition of ferric oxide onto the parent template of dolomite, albeit with reduced intensity. This reduction likely results from strong interactions between the metal particles and the support, potentially inducing structural changes because the Fe_2_O_3_ catalyst had the smallest crystallite size and resulted in the highest specific surface area among the metal oxide catalysts. Such interactions may partially disrupt the support framework, leading to reduced crystallinity or the formation of metal clusters or nanoparticles [26]. These effects can introduce strain and defects in the support material, further diminishing crystallinity. These peaks align with the XRD pattern of DM without significant deviation. However, the absence of distinct ferric oxide peaks, coupled with a reduction in the peak intensity of the XRD pattern for the DM template, suggests that the low ferric oxide content results in uniform dispersion or crystallite sizes smaller than 3 nm. Consequently, no significant changes in the chemical structure of the DM template are observed. A relationship exists between the catalyst crystallite size and specific surface area. These results are consistent with the fact that the wt.% of precursors incorporated during the preparation was sufficient for precipitation to occur. Overall, these findings suggest that wet impregnation of 5 wt.% Fe(NO_3_)_3_·9H_2_O onto dolomite does not significantly alter the structure or arrangement of the dolomite framework. Furthermore, the increase in ferric oxide content does not induce alterations in the chemical structure of the DM catalyst.

The catalyst characterization via X-ray diffraction (XRD) revealed that the chemical structure and crystallinity of ferric oxide in the catalyst could not be clearly identified. Therefore, X-ray fluorescence (XRF) was employed to determine the absorption of element-specific energy and confirm the sample’s elemental composition. Additionally, energy-dispersive X-ray fluorescence (EDXRF) was used to analyze the AC, DM, and Fe/DM catalysts, where varying Fe concentrations were introduced. This modification aimed to alter the acid strength over the active sites of the dolomite template. Table 2 presents the elemental analyses obtained by XRF, revealing that AC consists predominantly of carbon (98.73 wt.%), with trace amounts of other elements detectable in their oxide forms [21]. In contrast, calcined dolomite primarily comprises CaO (59.82 wt.%) and MgO (33.01 wt.%), which is consistent with the XRD diffractogram peaks corresponding to the crystallinity of CaO and MgO. Only a minor amount of ferric oxide (0.23 wt.% Fe_2_O_3_) was detected in the dolomite. For the Fe/DM catalysts, where 5 wt.% and 10 wt.% Fe(NO_3_)_3_·9H_2_O were incorporated into the dolomite template via wet impregnation, X-ray fluorescence (XRF) analyses confirmed Fe_2_O_3_ loadings of 5.82 wt.% and 9.14 wt.%, respectively, closely matching the intended impregnation concentrations.

Table 3 shows the EDXRF analyses of Fe_2_O_3_ at 0.04 wt.% in DM, which increased to 4.77 wt.% and 7.82 wt.% when 5 wt.% and 10 wt.% Fe were incorporated into the parent calcined dolomite, respectively. These results align well with the XRF pattern, confirming the efficacy of wet impregnation in uniformly dispersing Fe_2_O_3_ within the dolomite pore structure without significantly altering its XRD pattern compared with that of calcined dolomite. The absence of distinct Fe_2_O_3_ XRD peaks despite the 5–10 wt.% metal doping suggests either high dispersion of ferric oxide or the presence of crystallites smaller than 2 nm, which exhibit low-intensity peaks beyond the XRD detection limits.

**Figure 3:**
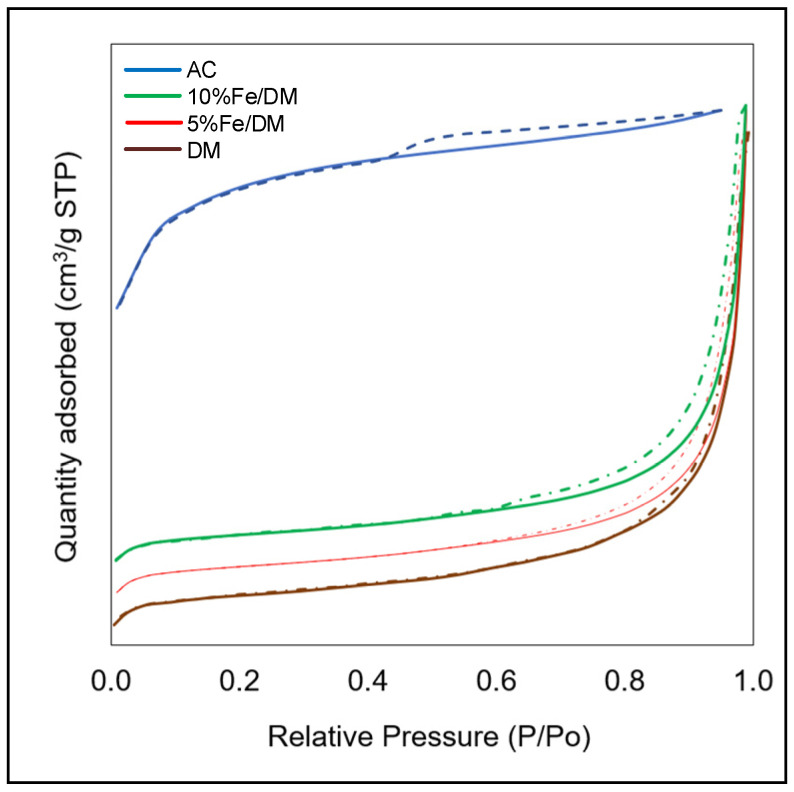
Adsorption and desorption isotherms.

The textural properties of the catalysts were investigated via nitrogen adsorption–desorption isotherms, which revealed a distinct hysteresis loop at high relative nitrogen pressures (P/P_0_), suggesting that the amounts of nitrogen gas adsorbed and desorbed are not equal, as shown in Figure 3. This suggests mesoporous structures (pore sizes between 2 and 50 nm) for AC, DM, 5 wt.% Fe/DM and 10 wt.% Fe/DM, and Table 4 summarizes the textural properties of the AC, DM, and Fe/DM catalysts, specifically the specific surface area and pore size of AC, calcined DM, 5 wt.% Fe/DM, and 10 wt.% Fe/DM.

The AC catalysts prepared from coconut shells have a specific surface area of 830.77 m^2^/g, a relatively small pore size of 2.37 nm, and a pore volume of 0.64 cm^3^/g. The Type IV isotherm with a hysteresis loop at a relative pressure (P/P0) of 0.44–0.89 confirms its mesoporous structure, in which AC has a high BET surface area, resulting in numerous acid strength active sites and a large total pore volume. Additionally, the consistent mesoporosity of AC, with abundant active sites and increased acid strength, influences its catalytic activity in C-C and C-H bond cleavage and aromatization reactions because of its pore selectivity and role as a catalyst in aromatization reactions [21,28]. The porosity of a catalyst greatly affects its catalytic activity, typically enhancing the delivery of the catalytic process to reactants. In contrast, the DM has mesopores with a lower porosity (0.10 cm^3^/g pore volume, 19.95 m^2^/g surface area).

The incorporation of 5 to 10 wt.% Fe into the dolomite template resulted in differences in specific surface area and pore size, resulting in the Fe/DM catalyst retaining mesoporous structures, as shown by the hysteresis loop observed at high nitrogen gas pressures during BET adsorption–desorption analysis, indicating that the amount of nitrogen gas adsorbed and desorbed is unequal to the type IV isotherms with a relative pressure (P/P0) of 0.42–0.96. A general trend was that increasing the ferric oxide loading led to a decrease in the specific surface area of the catalysts. Specifically, the 5 wt.% Fe/DM catalyst had a specific surface area of 18.77 m^2^/g and a pore size of 19.46 nm. The 10 wt.% Fe/DM catalyst further decreased the specific surface area (17.03 m^2^/g) and increased the pore size (25.80 nm). The incorporation of ferric oxide at 5 wt.% to 10 wt.% resulted in a decrease in both the specific surface area and pore volume while simultaneously increasing the pore size, which was likely due to the partial blockage of the dolomite template’s pore structure by a small amount of Fe_2_O_3_. Furthermore, the average pore size is a critical factor influencing the specific surface area.

The decrease in the specific surface area is attributed to the coverage of the catalyst surface by the Fe_2_O_3_ particles, resulting in a low specific surface area [18,21,26]. Additionally, the average pore size also plays a crucial role, with larger pores generally associated with lower specific surface areas. A catalyst with large pores generally has a lower specific surface area. However, if the pores are small, the specific surface area increases, which is in line with the impregnated catalyst with 5 wt.% to 10 wt.% ferric oxide incorporated into the parent calcined dolomite. This suggests that the rate of nucleation of Fe_2_O_3_ crystallites exceeds the rate of increase in the number of nuclei as the Fe loading to the dolomite parent template increases, leading to larger crystallite sizes and a corresponding gradual decrease in the surface area. However, the dispersion of metal oxides on the parent template is closely related to the acid strength of the active sites, as the acidity of a catalyst’s support affects its supporting properties upon modification during catalyst preparation. However, the acid strength active site of Fe/DM was not directly investigated via TPD-NH_3_ due to instrument limitations.

### 2.3. Univariate Experiment

A univariate analysis was conducted to investigate the effects of temperature and the N_2_ flow rate, as determined by the residence time and catalyst loading of the PPW feedstock, on the pyrolysis oil yield and product distribution, specifically that of the kerosene-like oil. This experiment utilized a catalyst loading of 5–20 wt.% (relative to the weight of PPW) and maintained constant across four catalyst types: AC, DM, 5 wt.% Fe/DM, and 10 wt.% Fe/DM. The systematic experimental design and response of the product distribution are presented in Table S1.

#### 2.3.1. Influence of the Catalyst Active Site

**Figure 4:**
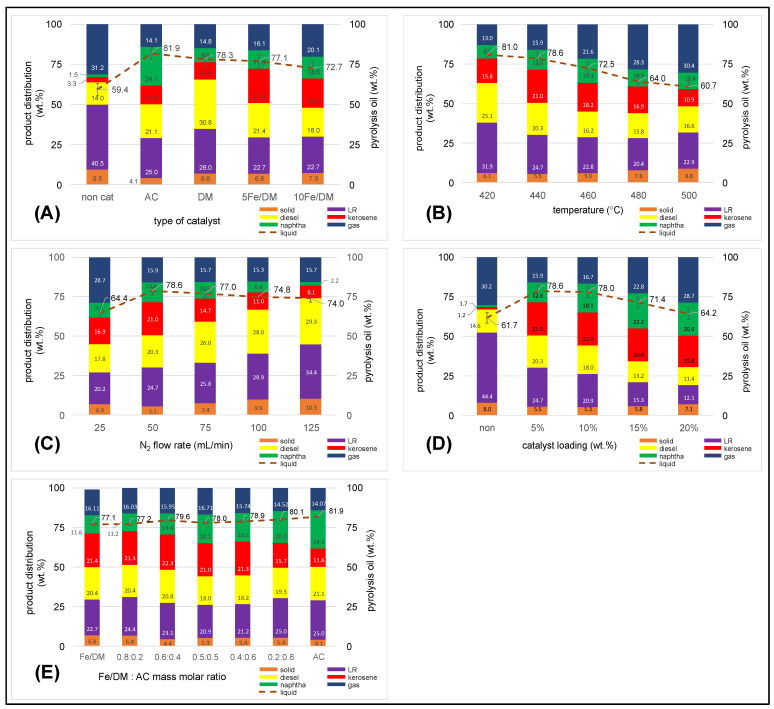
Influence of process parameters on product distribution: (**A**) influence of type of catalysis (**B**) influence of temperature; (**C**) influence of N_2_ flow rate; (**D**) influence of catalyst loading; (**E**) influence of Fe/DM:AC mass/molar ratio.

To investigate the catalytic active site when four catalyst types were used, AC, DM, 5 wt.%Fe/DM, and 10 wt.%Fe/DM were employed for the catalytic pyrolysis of PPW under operating conditions of 450 °C, with a N_2_ flow rate of 50 mL/min and a catalyst loading of 5 wt.% compared with a noncatalyst system. The product distribution, particularly the pyrolysis oil yield and kerosene-like yield, was also characterized to determine the influence of the acid strength active site in the catalysts used. Figure 4A shows the product distribution from noncatalytic pyrolysis of PPW, demonstrating the significant influence of temperature on the random scission of large hydrocarbon chains, which leads to the formation of free radicals, promoting β-scission reactions that yield shorter hydrocarbon radicals [12,14,29]. At high temperatures, the energy enthalpy significantly drives the random cleavage of these free radicals and hydrocarbon radicals into small, volatile vapors, which subsequently undergo secondary cracking reactions, which, in turn, cleave these volatile vapors into noncondensable light hydrocarbon gases under continued thermal decomposition. Simultaneously, some radicals are affected by temperature and break down into small noncondensable hydrocarbon gases and undergo polymerization reactions under high-temperature conditions, reforming longer hydrocarbon chains [30]. The product distribution analyses revealed yields of 31.2 ± 0.27 wt.% for noncondensable gases, 59.4 ± 0.26 wt.% for pyrolysis oil, and 9.5 ± 0.20 wt.% for solid residue. The high yields of solid residue are attributed to the influence of high temperature, which, upon thermal decomposition, results in both polymerized and secondary cracking reactions of small hydrocarbon radicals alongside the pyrolysis of large polymeric [30] and ad hoc components in the PPW feedstock that, upon thermal decomposition, form solid residues and polymerize into long-chain hydrocarbons. Simulated distillation gas chromatography of the pyrolysis oil indicated low yields of naphtha-like (1.5 ± 0.13 wt.%) and kerosene-like (3.3 ± 0.21 wt.%) fractions; notably, high-temperature-driven thermal cracking favored the breakdown of long-chain hydrocarbons into free and hydrocarbon radicals and suppressed hydrogen transfer reactions, resulting in C-C bond cleavage that would produce mid-chain hydrocarbons. Consequently, the mid-chain hydrocarbons produced initially further crack into smaller molecules and ultimately into noncondensable gases. Moreover, some hydrocarbon radicals undergo polymerization, forming medium-to-long hydrocarbon chains with a carbon range of C_19_^+^, with the heavy oil and LR fractions yielding 14.00 ± 0.16 wt.% and 40.5 ± 0.25 wt.%, respectively. These results suggest that thermal cracking alone induces random radical formation, producing significant noncondensable gas alongside repolymerized long-chain hydrocarbons. Dolomite (DM), a basic catalyst composed primarily of MgO, CaCO_3_, and CaO, promotes the thermal decomposition and catalytic cracking of large polymer chains [15,16]. The basic nature of DM facilitates the random thermal degradation of long polymer chains into smaller radicals, producing volatile vapors of suitable size to diffuse through the mesoporous structure and large surface area of the catalyst. The active sites, consisting of MgO and CaO on the catalyst surface [16,31], facilitate efficient C-H and C-C bond cleavage and subsequently accelerate the formation of small volatile vapors within the surface area and pore structure of the DM, enabling further isomerization and oligomerization reactions into light hydrocarbon chains [31,32], predominantly within the C_5_–C_19_ range. Figure 4A illustrates the performance of DM as a catalyst in PPW pyrolysis, where the reaction temperature enhances the thermal decomposition of the polymer chains and promotes the production of hydrogen and short-chain hydrocarbon radicals. These radicals can undergo hydrogenation on the MgO surface, enhancing product quality. The product distribution shows 14.8 ± 0.20 wt.% noncondensable gas, 78.3 ± 0.46 wt.% pyrolysis oil, and 6.9 ± 0.12 wt.% solid residue. This indicates that DM significantly promotes both thermal and catalytic activity through a mesopore structure. The catalytic activity of DM is crucial in facilitating C–C cleavage, enabling the conversion of volatile vapors into aliphatic light hydrocarbons, particularly in the 30.8 ± 0.11 wt.% diesel-like fraction (C_15_–C_19_ hydrocarbon chains) and the 28.0 ± 0.44 wt.% heavy long residue (LR). Fe/DM strongly enhances the effectiveness of the basic catalyst in enhancing both thermal and catalytic decomposition, yielding predominantly light hydrocarbons, supports the formation of diesel-range hydrocarbons, and the resulting product distribution includes only 8.8 ± 0.0.6 wt.% naphtha-like (C_5_–C_11_) and 10.8 ± 0.14 wt.% kerosene-like fractions. (C_11_–C_15_) because its selectivity for lighter fractions remains limited under the basic catalysts and mesopore structures with large surface areas [15,31].

As shown in Figure 4A, the influence of the AC catalyst on the catalytic activity is illustrated with the AC catalyst employed in the catalytic pyrolysis of PPW. The product distribution analysis revealed that the yield of the noncondensable material was only 14.1 ± 0.14 wt.%, with solid residue and carbonaceous material at 4.1 ± 0.08 wt.% and a pyrolysis oil yield of 81.9 ± 0.45 wt.%. This finding demonstrated the significant influence of temperature on the random cleavage of C-C and C-H bonds in large hydrocarbon chains in forming a mid-hydrocarbon radical. The use of AC, which is characterized by acid active sites with relatively micro- and meso-porous properties, a large specific surface area, a large pore volume, and a wide pore size distribution, significantly enhances accelerated C-C bond cleavage [30]. As the temperature increased during catalytic pyrolysis, the acidic strength of the large surface area, coupled with the porosity of the AC catalyst, enhanced facilitated C-C bond cleavage. This result led to the formation of smaller hydrocarbon compounds through reactions at the acid active sites and within the mesopores of the AC catalyst. The cleavage of C-C bonds subsequently occurs via hydrogen transfer reactions on the surface of the AC catalyst [29,30]. The increased acid strength of the AC catalyst further promoted C-C bond cleavage through β-scission, which resulted in the breakdown of long hydrocarbon chains and their conversion to shorter hydrocarbon chains. Simultaneously, the pore distribution and large surface area of the AC catalyst facilitate hydrogen transfer and hydrogenation reactions of shortened hydrocarbon chains at the acid active sites. These reactions enabled pore-selective reactions, isomerization, oligomerization, and aromatization of shorter hydrocarbons consisting mainly of light aliphatic hydrocarbons, yielding a naphtha-like fraction that reached a maximum of 24.1 ± 0.19 wt.% and kerosene-like fractions of 11.6 ± 0.23 wt.%. During the catalytic pyrolysis of PPW, the operating temperature drives thermal cracking into mid-hydrocarbon chains. The AC catalyst significantly promoted the conversion of long hydrocarbon chains, resulting in a reduction in the heavy long residue fraction to 25.0 ± 0.0.27 wt.%, leading to production in the diesel-like range at 21.1 ± 0.21 wt.%. The AC catalyst was highly effective in the catalytic cracking of mid-sized hydrocarbons through hydrogenation, isomerization, oligomerization, and aromatization reactions, yielding up to 24.1 ± 0.19 wt.% naphtha-like products. Moreover, smaller hydrocarbon compounds undergo continuous cracking, forming noncondensable gases and small hydrocarbon gases.

The incorporation of varying concentrations of Fe_2_O_3_ (5 wt.% and 10 wt.%) into DM catalysts significantly enhances the catalytic activity by increasing the acid strength of the active sites, surface area and pore volume of the DM catalyst when conducted at 450 °C under a nitrogen gas flow rate of 50 mL/min with 5 wt.% catalyst loading relative to the feedstock. The yields of the products, including noncondensable gases, pyrolysis oil, and solid residue (both carbonaceous and other residual solids remaining from pyrolysis), are shown in Figure 4A. Notably, when the 5 wt.% Fe/DM catalyst was used, the product distribution consisted of 16.1 ± 0.14 wt.% noncondensable gases, 78.1 ± 0.25 wt.% pyrolysis oil, and 6.8 ± 0.07 wt.% solid residue, causing a uniform dispersion of Fe_2_O_3_ on the pore surface area, and the pore volume of the DM template increased the acid strength of the active sites on the Fe/DM and facilitated hydrogen transfer and β-scission of the C-H and C-C bonds. This leads to the formation of shorter volatile vapors, which promote hydrogenation, isomerization, and oligomerization reactions that accelerate the conversion of pyrolysis oil into noncondensable gases [26,27,33,34]. When the concentration of Fe_2_O_3_ on the DM template with the 10 wt.% Fe/DM catalyst is increased, the pyrolysis oil yield decreases to 72.7 ± 0.24 wt.%, which is further subjected to a catalytic cracking reaction driven by the acid active sites of the Fe-impregnated DM template, resulting in the cleavage of C-C bonds and C-H bonds to form shortened volatile vapors, whereas higher Fe_2_O_3_ concentrations cause Fe_2_O_3_ particles to block active sites and reduce the pore volume, favoring thermal cracking over catalytic cracking. This promotes random thermal cleavage into small free radicals and hydrocarbon radicals at high temperatures, which undergo secondary cracking reactions, producing noncondensable gases and contributing to catalyst deactivation [31,33,34]. Consequently, a decreasing pyrolysis oil yield and increasing noncondensable gas yield are observed.

#### 2.3.2. Influence of Temperature

To investigate the effect of temperature on the pyrolysis of PPW, a semi-batch reactor was used under the following process conditions: an inert N_2_ flow rate of 50 mL/min and a blending of AC with 5 wt.% Fe/DM in a 0.5:0.5 mass/molar ratio, and a catalyst loading of 5 wt.% relative to the PPW feedstock. Generally, pyrolysis is driven primarily by thermal A decomposition. Therefore, temperature is a critical parameter for thermal cracking. As the temperature increases, the thermal cracking reaction initiates the random breakdown of large hydrocarbon molecules, which are composed mainly of carbon, hydrogen, and oxygen, via C-C and C-H bond cleavage. Figure 4B illustrates the product distribution from the catalytic pyrolysis of PPW at various pyrolysis temperatures. Increasing the process temperature accelerates the random cleavage of C-C bonds from van der Waals forces along the hydrocarbon chain, which are greater than the enthalpy of the C-C bonds, and the randomized thermal degradation of these polymers is accelerated [12,14,32], resulting in the production of small volatile vapors. Notably, as increasing the temperature from 420 °C to 500 °C monotonically decreases the pyrolysis oil yield from 81.0 ± 0.48 wt.% to 60.7 ± 0.52 wt.%, a statistically significant decline in the pyrolysis oil yield is observed at 500 °C compared with that at 480 °C (64.0 ± 0.31 wt.%, *p* < 0.05), which is consistent with secondary cracking mechanisms dominating above 480 °C. This phenomenon is consistent with the effect of high temperature, which causes the small volatile vapor to undergo further degradation through both thermal decomposition and secondary cracking reactions, resulting in the formation of noncondensable gases. During pyrolysis, temperature primarily induces random thermal decomposition of PPW into free radicals, followed by depolymerization into smaller hydrocarbon radicals through β-scission. This stage degrades large hydrocarbon chains into medium-sized and shortened hydrocarbons. Notably, high temperatures significantly affected the secondary decomposition of volatile hydrocarbon compounds, further reducing the pyrolysis oil yield as the temperature increased from 460 °C to 500 °C, resulting in extremely low pyrolysis oil yields from 72.5 ± 0.39 wt.% to 60.7 ± 0.52 wt.%. At these higher temperatures, the yield of noncondensable compounds increased monotonically from 13.0 ± 0.21 wt.% to 30.4 ± 0.12 wt.% as the temperature increased from 420 °C to 500 °C. Conversely, the LR yield decreased from 31.9 ± 0.35 wt.% to 20.4 ± 0.33 wt.% between 420 °C and 480 °C, but notably, the LR yield increased slightly to 22.9 ± 0.23 wt.% at 500 °C, resulting in an increase in carbonaceous and solid residue from 5.9 ± 0.09 wt.% to 8.8 ± 0.19 wt.% as the temperature increased from 460 °C to 500 °C. Notably, when the pyrolysis temperature increases to 500 °C, the gas yield also increases because high temperatures promote C-C bond cleavage, resulting in the production of a large amount of free radicals that shorten hydrocarbon compounds in volatile vapors. These reactions promote thermal degradation into smaller hydrocarbon vapors via secondary cracking reactions and accelerate the degradation of hydrocarbon chains into smaller radicals and, consequently, into noncondensable gases, increasing the noncondensable gas yield [32,33,34].

In contrast, the pyrolysis oil yield remains stable between the thermal decomposition of pyrolysis at 420 °C and 460 °C, i.e., 81.0 ± 0.48 wt.% and 72.5 ± 0.51 wt.%, respectively, suggesting minimal thermal degradation of volatile vapors in this range. The moderate temperature enhanced heat transfer, promoting random C-C bond cleavage, and subsequent β-scission, which broke down larger hydrocarbons into middle hydrocarbons and enhanced the cleavage of C-C bonds into shortened volatile components. These volatile vapors then undergo further thermal decomposition and secondary cracking reactions, favoring the formation of smaller hydrocarbons and noncondensable gases without significant polymerization reactions.

The catalytic role of Fe/DM is as a dual-function catalyst featuring acid strength active sites on dolomite-supported basic catalysts with mesoporous properties, whereas AC catalysts contribute acid active sites across a high surface area and pore-selective properties. These characteristics increase the catalytic activity, resulting in the catalytic cracking of medium hydrocarbons into shorter hydrocarbon chains. The active sites facilitate the hydrogen transfer reaction, enabling diffusion in both the micro- and mesopores of the Fe/DM and AC catalysts [21,26,28]. The textural properties of AC, characterized by small pore sizes and large surface areas, enhance its pore selectivity toward C_5_–C_15_ volatile vapors. These vapors subsequently undergo hydrogenation, isomerization, and aromatization, forming light aliphatic hydrocarbons in the C_11_–C_15_ range. The product distribution of pyrolysis oil corresponds to the kerosene-like fraction based on the crude oil boiling point temperature range determined by a gas chromatograph that simulates distillation according to the ASTM D86 standard.

Between 420 °C and 440 °C, the yields of the kerosene-like and naphtha-like fractions increase from 15.6 ± 0.11 wt.% to 21.0 ± 0.49 wt.% and from 8.5 ± 0.29 wt.% to 12.6 ± 0.23 wt.%, respectively. This suggests a synergistic effect between thermal cracking and catalytic cracking, converting larger hydrocarbon chains into mid-range hydrocarbons. This conversion involves free radicals, C-C bond scission, hydrogen transfer, and catalytic activity, including hydrogenation, isomerization, and aromatization, facilitating hydrocarbon structural rearrangement into aliphatic hydrocarbons with a carbon range of C_11_–C_15_ [9]. At temperatures ranging from 460 °C to 500 °C, thermal cracking dominated over catalytic cracking. This leads to increased random thermal degradation and accelerated cracking of small volatile vapors, yielding noncondensable gases through randomized C-C bond cleavage and the production of numerous free radicals that form small hydrocarbon compounds. The noncondensable gas yield increases from 21.6 ± 0.12 wt.% to 30.4 ± 0.12 wt.%, whereas the pyrolysis oil yield decreases from 72.5 ± 0.61 wt.% to 60.7 ± 0.33 wt.%. The kerosene-like fraction also decreases from 18.2 ± 0.24 wt.% to 10.9 ± 0.34 wt.% when the temperature increases from 460 °C to 500 °C. Simultaneously, the thermal decomposition of volatile vapors decomposes into small hydrocarbon radicals, and therefore, the oligomerization and polymerization of mid-range hydrocarbon chains contribute to the formation of heavier hydrocarbon compounds. This increases the long-residue hydrocarbon (C_19_^+^) yield from 20.4 ± 0.33 wt.% to 22.9 ± 0.23 wt.% as the temperature increases from 480 °C to 500 °C, resulting in accelerated polymerization of small volatile vapors, which may occur to produce large polymers or long hydrocarbon chains such as waxes [30,34], which bind with carbonaceous and solid residues. Therefore, the solid residue and LR fractions increase, with LR yields of 20.4 ± 0.33 wt.% and 22.9 ± 0.23 wt.% and solid residue yields of 7.8 ± 0.17 wt.% and 8.8 ± 0.19 wt.% at pyrolysis temperatures of 480 °C and 500 °C, respectively, resulting in a reduced pyrolysis oil yield, with light aliphatic hydrocarbon fractions, including kerosene-like (10.9 ± 0.34 wt.%) and naphtha-like (10.4 ± 0.24 wt.%), decreasing at pyrolysis temperatures of 500 °C.

#### 2.3.3. Influence of the Inert N_2_ Flow Rate

Figure 4C shows the product distribution when the catalytic pyrolysis of PPW was performed under the following process conditions: 440 °C, a blending of AC with 5 wt.% Fe/DM in a 0.5:0.5 mass/molar ratio, and a catalyst loading of 5 wt.% relative to the PPW feedstock by varying the inert N_2_ flow rate between 25 mL/min and 125 mL/min. Generally, catalytic pyrolysis with a carrier gas feed is employed to regulate the residence time of reactants within the reactor, which is typically controlled by adjusting the gas flow rate, resulting in the product distribution. The process temperature plays a crucial role in driving the thermal decomposition of long-chain hydrocarbon compounds, particularly those leading to the formation of free radicals and hydrogen radicals, which undergo C-C bond and C–H bond cleavage via β-scission reactions, leading to shortened hydrocarbon vapor before the catalyst facilitates further cracking reactions [12,14,30,32]. The catalyst plays a role in decomposition at either acidic or basic active sites, and β-scission reactions reduce the hydrocarbon chain length. The subsequent catalytic cracking via the hydrogen transfer reaction accelerated the decomposition of volatile vapors, whereas the catalytic activity of subsequent reactions, such as hydrogenation, isomerization, oligomerization and aromatization, depended on the textural properties of the catalyst, which also properly produced light aliphatic hydrocarbon compounds [9,35,36].

When inert N_2_ at a low flow rate serves as a means to regulate the residence time of the pyrolysis reaction, it provides extended residence times for thermal cracking and catalytic cracking. The thermal decomposition reaction accelerated random C-C bonds and C-H bonds during large hydrocarbon changes, resulting in the formation of free and hydrocarbon radicals. At a nitrogen (N_2_) flow rate of 25 mL/min, a low flow rate extends the residence time of volatile vapors, allowing sufficient hydrocarbon radicals to derive from thermal cracking and subsequently facilitating the hydrogen transfer of volatile vapors with acid strength active sites on the Fe/DM catalyst. The acidic active site and large surface area of the mesoporous DM support also promote catalytic cracking and further secondary cracking reactions, such as hydrogenation, isomerization, and oligomerization, ultimately yielding light hydrocarbon vapors before being carried out of the reactor by the swept N_2_ gas. The combined acidic active site and micro/mesoporous structure of the AC catalyst, characterized by a high surface area, promotes hydrogenation, isomerization, oligomerization, and aromatization [21,29,30], leading to the formation of light hydrocarbon vapors, which are then carried out in the reactor by nitrogen flow. In contrast, excessively long residence times can lead to the continued degradation of these volatile vapors to secondary cracking reactions driven by prolonged exposure to high temperatures and catalytic effects, resulting in random thermal degradation and a secondary cracking reaction favoring the formation of noncondensable gases [32,33].

Moreover, extended high-temperature conditions promote the formation of solid residues and excessive carbonaceous deposits, which can cover the acid strength active sites of the Fe/DM catalyst, including accumulated blockage within the pore structure of both Fe/DM and AC, resulting in catalytic deactivation coupled with the polymerization of volatile vapors into heavy long-chain hydrocarbons under these conditions, which is consistent with the product distribution, which is represented by a greater yield of noncondensable gas. LR and solid residue are shown in Figure 4C. At a N_2_ flow rate of 25 mL/min, yields of 28.7 ± 0.12 wt.% noncondensable gas, 64.4 ± 0.35 wt.% pyrolysis oil, and 6.9 ± 0.12 wt.% solid residue were observed. A Sim-DGC analyses according to ASTM D86 of the pyrolysis oil reveal 9.6 ± 0.22 wt.% naphtha-like, 16.9 ± 0.19 wt.% kerosene-like, 17.8 ± 0.18 wt.% diesel-like, and 20.2 ± 0.32 wt.% LR fractions.

Increasing the nitrogen flow rate to 50 mL/min resulted in a significant shift in the product distribution of the pyrolysis oil to 78.6 ± 0.25 wt.%, and the noncondensable gas content dramatically decreased to 15.9 ± 0.13 wt.%, whereas the solid residue content slightly decreased to 5.5 ± 0.07 wt.%. Notably, the product distribution of pyrolysis oil via Sim-DGC analysis revealed increases in both kerosene-like (21.0 ± 0.49 wt.%) and naphtha-like (12.6 ± 0.23 wt.%). However, the product distribution yielded a 20.3 ± 0.34 wt.% diesel-like fraction and 24.7 ± 0.25 wt.% LR fraction. On the other hand, high N_2_ flow rates reduce the residence time, shorten and limit the thermal decomposition of the mid-hydrocarbon chain, which is influenced by both the temperature and the reactor being carried out by the swept N_2_ gas. The thermal decomposition of PPW largely occurs via free radical reactions, hydrocarbon radicals, and further decomposition of hydrocarbon radicals into mid-hydrocarbon chains. This short residence time is insufficient to fully break down large hydrocarbon chains, limiting the opportunity for mid-hydrocarbon radicals to undergo further cracking or remain too large to diffuse through the mesoporous structure [35]. However, temperature effectively induces further C-C bond and C–H bond cleavage into small chains at the acid active site and surface pore structure of both the Fe/DM and AC catalysts [21,28,31,36]. However, the short residence time was also not sufficient to induce bond cleavage of volatile vapors, resulting in their inability to effectively pass through the mesoporous structure of the DM and AC catalysts. As a result, the amount of mid-volatile vapor remaining too large to diffuse through the catalyst active sites does not increase the catalytic activity, including hydrogenation, isomerization, and oligomerization reactions, and limits the extent of the secondary cracking reaction of the shortened volatiles, thereby reducing the production of desirable light aliphatic hydrocarbons. Consequently, the resulting pyrolysis oil with a high N_2_ flow rate due to the short residence time prevents large hydrocarbon molecules from diffusing through the mesoporous structure of the DM from reacting at catalytically active sites [31], resulting in the pyrolysis oil retaining a high proportion of the LR fraction, while a slight increase in the solid residue and carbonaceous materials was also observed.

A further increase in the nitrogen flow rate to 75, 100, and 125 mL/min did not significantly alter the yield of noncondensable gases, which remained relatively stable between 15.3 ± 0.10 wt.% and 15.7 ± 0.13 wt.%. The pyrolysis oil yield slightly decreased from 77.0 ± 0.29 wt.% to 74.0 ± 0.38 wt.%, and the solid residue yield increased from 7.4 ± 0.06 wt.% to 10.3 ± 0.07 wt.%. The Sim-DGC analyses revealed that at 125 mL/min, the pyrolysis oil had the highest LR fraction of 34.4 ± 0.47 wt.%, followed by the diesel-like fraction of 29.3 ± 0.18 wt.%, the kerosene-like fraction of 8.1 ± 0.12 wt.%, and only the naphtha-like fraction of 2.2 ± 0.09 wt.%, indicating inefficient thermal and catalytic cracking at short residence times. Therefore, the N_2_ flow rate significantly influences the residence time and product distribution in catalytic pyrolysis and, consequently, the extent of secondary reactions and product selectivity. Low N_2_ flow rates increase light hydrocarbon production, but further secondary cracking reactions produce abundant noncondensable gases and catalyst deactivation due to prolonged residence times. High N_2_ flow rates limit the decomposition of large molecules, resulting in higher LR fractions and solid residues.

#### 2.3.4. Influence of Catalyst Loading

This study investigated the different ratios of a dual catalyst (iron-doped dolomite (Fe/DM) and activated carbon (AC)) affecting the production distribution. The catalyst loading to feedstock mass ratio varied from 5 wt.% to 20 wt.%. The experiments were conducted at 440 °C with a nitrogen flow rate of 50 mL/min and kept constant at 0.5:0.5 of Fe/DM:AC. The results were compared to those of noncatalytic pyrolysis under the same conditions. The key finding was that catalyst loading into the feedstock significantly impacts the product distribution of pyrolysis products. In the absence of a catalyst, as depicted in Figure 4D, the temperature and residence time significantly influenced the thermal decomposition of PPW. This observation aligns with the thermogravimetric analysis (TGA) and derivative thermogravimetric (dTG) results, which revealed that substantial weight loss occurred between 300 and 450 °C. Thermal degradation involves the cleavage of C–C and C–H bonds within long hydrocarbon chains, resulting in free radicals. These radicals underwent rearrangement to form shorter hydrocarbons and further secondary cracking reactions that converted volatile vapors into hydrogen (H_2_) and other noncondensable gases [6,7,32], yielding a high yield of noncondensable gas of 30.2 ± 0.10 wt.%. The solid residue, accounting for 8.0 ± 0.03 wt.%, resulted from the polymerization of hydrocarbon radicals and mid-length hydrocarbon chains into longer hydrocarbon chains at elevated temperatures [7,32], along with the deposition of carbonaceous materials during the pyrolysis reaction.

Noncatalytic pyrolysis of PPW yielded 61.7 ± 0.52 wt.% pyrolysis oil. Analysis of this pyrolysis oil via simulated distillation gas chromatography (Sim-DGC) revealed that the pyrolysis oil predominantly comprised a long residue (LR) fraction of up to 44.4 ± 0.17 wt.%. A high LR fraction was associated with an increased solid residue fraction through polymerization reactions [30,32,35]. The remaining pyrolysis oil consisted of a diesel-like fraction at 14.6 ± 0.11 wt.%, with minor contributions from the kerosene-like (1.7 ± 0.17 wt.%) and naphtha-like (1.2 ± 0.14 wt.%) fractions. The low proportion of kerosene-like and naphtha-like fractions resulted from the thermal degradation and random cleavage of C–C and C–H bonds, which produced mid-length hydrocarbon radicals. These radicals underwent a secondary cracking reaction, resulting in smaller, more volatile vapors, which either decomposed into noncondensable gases or repolymerized into larger hydrocarbon chains, contributing to the elevated LR fraction and solid residue amounts.

When the catalyst loading-to-feedstock ratio was varied from 5 wt.% to 20 wt.%, the addition of 5 wt.% Fe/DM blended with the AC catalyst (Fe/DM-AC) significantly influenced the pyrolysis product distribution yield. Increasing the catalyst loading from 5 wt.% to 10 wt.% resulted in a slight increase in the amount of noncondensable gas present, which ranged from 15.9 ± 0.13 wt.% to 16.7 ± 0.21 wt.%. The trend shows a dramatic increase in noncondensable gas to 22.8 ± 0.15 wt.% and 28.7 ± 0.17 wt.% when the catalyst loading increases from 15 wt.% to 20 wt.%. The highest noncondensable gas yield occurred when the Fe/DM-AC catalyst was loaded with feedstock at 20 wt.%, which was similar to the results of noncatalytic pyrolysis. Additionally, the yields of the solid residue and carbonaceous material also tended to increase slightly from 5.5 ± 0.07 wt.% to 5.8 ± 0.11 wt.% when the catalyst loading increased from 5 wt.% to 15 wt.%, and a more significant increase to 7.1 ± 0.08 wt.% was observed when the catalyst loading increased to 20 wt.%. This trend aligns with the catalytic activity of the dual Fe/DM-AC catalyst, driven by the acid strength active sites of Fe_2_O_3_ on dolomite and the influence of the pore-selective structure and high surface area of the AC catalyst. Therefore, the pyrolysis oil yield monotonically decreased from 78.6 ± 0.62 wt.% to 64.2 ± 0.51 wt.% when the catalyst loading of the feedstock increased from 5 wt.% to 20 wt.%. The experimental results indicate that temperature affects the thermal decomposition of PPW, initiating random C-H and C-C bond cleavage, resulting in the degradation of large hydrocarbons, and leading to the production of free radicals and hydrocarbon radicals. These further degrade into mid-volatile vapor through hydrogen transfer and C-C bond cleavage via β-scission, yielding mid-hydrocarbon chains. The catalytic activity of dual Fe/DM-AC enhances accelerated hydrogen transfer at acid strength-activated sites on the high surface area of dolomite. This accelerates β-scission and C-C bond cleavage, promoting the decomposition of mid-hydrocarbons into straight aliphatic hydrocarbons at those acid strength active sites. The micro/mesopore structure and large surface area of the AC catalyst continue to enhance the catalytic activity and secondary cracking reactions to obtain short, straight aliphatic hydrocarbons. Consequently, as catalyst loading increases, the concentration of acidic active sites increases, promoting further hydrogenation, isomerization, oligomerization, and aromatization reactions and selectively converting condensable volatiles, leading to the formation of shorter straight-chain aliphatic hydrocarbons [9,21,27,28,34].

At high catalyst loadings, particularly 20 wt.%, secondary cracking reactions become significant, greatly influence cracking into straight aliphatic hydrocarbons, and are influenced by the secondary cracking reaction. These shortened straight aliphatic hydrocarbons further decompose into noncondensable gases, along with carbonaceous deposits at the acidic active sites of the catalyst that cause pore blockage, leading to catalyst deactivation [32,33,34,35]. This explains the substantial increase in noncondensable gas and solid residue yields when the catalyst loading was increased to 20 wt.%.

Product distribution analyses Sim-DGC revealed that at 5 wt.% catalyst loading, a 24.7 ± 0.25 wt.% LR fraction, 20.3 ± 0.34 wt.% diesel-like fraction, a 21.0 ± 0.12 wt.% kerosene-like fraction, and a 12.6 ± 0.23 wt.% naphtha-like fraction were obtained. Increasing the catalyst loading to 10 wt.% and 20 wt.% led to a significant decrease in the diesel-like and LR fractions, which decreased to 11.4 ± 0.31 wt.% and 12.1 ± 0.18 wt.%, respectively. A maximum kerosene-like yield of 21.0 ± 0.28 wt.% was obtained with 10 wt.% loading catalyst, with an insignificant change observed upon the catalyst loading from 10 wt.% to 20 wt.%. Notably, the highest naphtha-like yield of 22.2 ± 0.20 wt.% was achieved at 15 wt.% catalyst loading, followed by a slight decrease to 20.9 ± 0.17 wt.% when the catalyst loading to feedstock at 20 wt.%

These results align with the acid strength active sites on the Fe/DM-AC catalyst surface and its textural structure, which promote catalytic reactions such as hydrogen transfer and hydrogenation of mid-volatile vapors, which are enhanced with increasing catalyst loading [6,37], leading to continuous thermal degradation of mid-volatile vapors through hydrogen transfer and hydrogenation at the acid strength active sites of both the DM and AC catalysts. In particular, the pore-selective properties of micro- and mesopores within the high surface pore structure facilitate the diffusion of volatile vapors to enable catalytic cracking because the textural properties of the catalyst result in isomerization, oligomerization, and aromatization reactions, effectively cracking large hydrocarbon chains into mid-hydrocarbons, which are then converted to shorter straight-chain aliphatic hydrocarbons at Fe/DM-AC dual catalyst loadings between 5 wt.% and 10 wt.%.

The LR yield fraction decreases from 24.7 ± 0.07 wt.% to 20.9 ± 0.22 wt.%, 15.3 ± 0.35 wt.%, and 12.01 ± 0.18 wt.% as the catalyst loading increases from 5 wt.% to 20 wt.%. The diesel-like fraction follows a similar declining trend, suggesting that the thermal degradation of large hydrocarbon chains is significantly influenced by the abundant acidic active sites on the large surface area of the DM catalyst support, facilitating catalytic cracking and promoting hydrogen transfer, hydrogenation, isomerization, oligomerization, and aromatization reactions, ultimately yielding shorter straight aliphatic hydrocarbons in the C_5_-C_11_ (naphtha-like) and C_11_-C_15_ (kerosene-like) carbon ranges.

However, increasing the catalyst loading to the feedstock at 20 wt.% led to a greater abundance of acidic active sites, which caused the hydrogen transfer reactions at the active sites to degrade middle hydrocarbons into shortened aliphatic hydrocarbons. Notably, the conversion of intermediate hydrocarbon compounds into shortened aliphatic hydrocarbons did not significantly increase, even when a catalyst loading of 20 wt.%, was used because the high catalyst loading could be attributed to excessive secondary cracking with a high number of acid active sites, facilitating the continuous degradation of the C-C bonds of small aliphatic hydrocarbons into noncondensable gases. Notably, high catalyst loading accelerated the catalytic activity, causing hydrogen transfer reactions along with hydrogenation reactions, which increased the ability of C-C and C-H bond scissions, accelerating the continuing degradation of light compounds into noncondensable gas products in greater proportions and further influencing the secondary cracking reaction that decomposes volatile vapor into noncondensable gas [33,34].

Excessive catalyst loading (20 wt.% dual Fe/DM-AC) was less beneficial and did not favor the production of desirable shortened straight-chain aliphatic hydrocarbons in the kerosene- and naphtha-like ranges. Instead, it increases noncondensable gas and solid residue yields due to catalyst deactivation and a reduced liquid yield. Thus, optimizing catalyst loading is critical for maximizing the production of valuable liquid hydrocarbons while minimizing unwanted byproducts during the catalytic pyrolysis of PPW.

#### 2.3.5. Influence of a Dual Catalyst at Different Mass Ratios of Fe/DM and AC Catalysts

The effects of varying the mass/molar ratio of Fe/DM blended with AC catalysts, ranging from 0.8:0.2 to 0.2:0.8, on the product distribution from the catalytic pyrolysis of PPW were investigated under operating conditions of 440 °C, a nitrogen gas flow rate of 50 mL/min, and a constant catalyst-to-feedstock loading of 10 wt.%. The results were compared to those obtained using individual catalysts under identical conditions, as shown in Figure 4E. Univariate experiments on the effect of catalytic active sites revealed a significant influence of temperature on the random scission of large hydrocarbon chains, resulting in the formation of free radicals, which promote β-scission reactions that yield shorter hydrocarbon radicals [6,32]. Concurrently, the radicals interact with the acidic active sites, and hydrogen transfer leads to the formation of smaller volatile vapors, followed by hydrogenation, isomerization, oligomerization, and aromatization [21,27,28]. These reactions shorten straight aliphatic hydrocarbons. The catalytic performance and surface pore structure of a catalyst are crucial factors in this process. Specifically, the Fe/DM catalysts provide acidic active sites via iron doping of the dolomite template, whereas dolomite acts as a basic catalyst that enhances both thermal decomposition and catalytic cracking [15,16,31], predominantly yielding light hydrocarbons. Moreover, the AC catalyst, with its strong acid active sites and micro/mesoporous structure, accelerates the cleavage of mid-volatile vapors into smaller volatile vapors. Further reactions, coupled with catalytic activity via hydrogenation, isomerization, oligomerization, and aromatization to yield shortened straight aliphatic hydrocarbons in the C_5_–C_11_ and C_11_–C_15_ carbon ranges, are promoted. Therefore, combining Fe/DM with AC was used to investigate the product distribution and identify the conditions yielding the highest proportion of the desirable kerosene-like fraction.

When PPW was pyrolyzed with only the AC catalyst, the process yielded 14.1 ± 0.11 wt.% noncondensable gas, 81.9 ± 0.15 wt.% pyrolysis oil, and 4.1 ± 0.07 wt.% solid residue. Product distribution analysis of the pyrolysis oil according to ASTM D86 standards revealed naphtha-like (24.1 ± 0.21 wt.%), kerosene-like (11.6 ± 0.16 wt.%), and diesel-like (21.1 ± 0.28 wt.%). The pyrolysis of PPW with the 5 wt.% Fe-doped DM catalyst produced more noncondensable gas (16.1 ± 0.12 wt.%), along with 11.6 ± 0.11 wt.% naphtha-like, 21.4 ± 0.27 wt.% kerosene-like, 20.4 ± 0.16 wt.% diesel-like, 22.7 ± 0.35 wt.% LR, and 6.8 ± 0.08 wt.% solid residue.

When the dual Fe/DM-AC catalyst is used at a 0.8:0.2 mass/molar ratio, the catalytic activity of both Fe/DM, which illustrates acidic active sites on a mesoporous structure with a large surface area, and AC, which represents strongly acidic active sites with micro/mesoporous pore surface structures, is combined. This combination yielded 16.0 ± 0.13 wt.% noncondensable gas, 77.2 ± 0.18 wt.% pyrolysis oil, and 6.8 ± 0.07 wt.% solid residue, with the pyrolysis oil consisting of 11.2 ± 0.19 wt.% naphtha-like, 21.3 ± 0.19 wt.% kerosene-like, 20.4 ± 0.37 wt.% diesel-like, and 24.4 ± 0.26 wt.% LR fractions. Increasing the AC proportion from 0.8:0.2 Fe/DM-AC to 0.2:0.8 Fe/DM-AC mass/molar ratios tended to increase the naphtha-like content from 11.2 ± 0.16 wt.% to 20.2 ± 0.18 wt.%, with a corresponding decrease in the kerosene-like content from 21.3 ± 0.19 wt.% to 15.7 ± 0.24 wt.%. However, the diesel-like and LR fractions did not significantly change across the different dual Fe/DM-AC catalyst ratios. The diesel-like did not significantly change with variations in the Fe/DM-AC ratio. This reaction can be attributed to the influence of thermal degradation, leading to random scission of C-C and C-H bonds, which is accelerated to form free radicals and hydrocarbon radicals. These radicals promote C-C bond cleavage via the β-scission of large hydrocarbon compounds, which are then converted into mid-volatile vapors with subsequent scission to produce smaller volatile vapors that are still large hydrocarbon compounds that cannot react with the acid active sites, diffuse through the small pore structures, and undergo further reactions [21,26,31].

**Figure 5:**
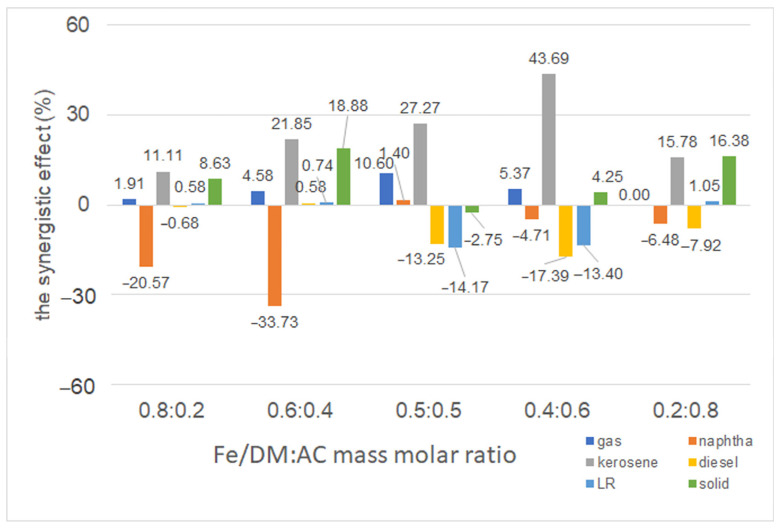
Synergy of the mass/molar ratio of the Fe/DM-AC catalyst.

Therefore, the use of an Fe/DM catalyst with acidic active sites and a mesoporous pore surface structure induces a reaction through its catalytic activity to convert it into a smaller volatile vapor, which is small enough to diffuse into the catalyst. In contrast, when a 0.2:0.8 mass ratio of Fe/DM and AC catalysts is used, the mid-volatile vapor generated from thermal decomposition by free radicals and hydrocarbon radicals is still too large to diffuse into the micropore surface structure; however, the AC catalyst still has more acidic active sites than the DM catalyst does [16,21,31,34], enabling catalytic reactions such as hydrogenation, isomerization, oligomerization, and aromatization. The results revealed that the catalytic pyrolysis of the PPW reaction was induced through the catalytic activity of the AC catalyst, which changed the intermediate volatile vapor, to shorten the volatile vapor to naphtha, similar to C_5_–C_11_, rather than kerosene-like (C_11_–C_15_). The results demonstrated that the pyrolysis oil yield when the 0.6:0.4 Fe/DM-AC mass ratio was used yielded 79.6 ± 0.35 wt.% pyrolysis oil, which was slightly greater than the 77.2 ± 0.22 wt.% yield achieved with a 0.8:0.2 ratio of the Fe/DM-AC mass ratio. Nevertheless, the highest kerosene-like fraction (22.3 ± 0.22 wt.%) was obtained with the 0.6:0.4 Fe/DM-AC mass/molar ratio. Therefore, to further understand the synergistic effects of blending AC with Fe/DM at different ratios, additional investigations were conducted, and variations in their blending ratios were systematically investigated [38,39,40], as depicted in Figure 5. The experiments were conducted using both individual and dual catalysts under the following operating conditions: 440 °C, a N_2_ flow rate of 50 mL/min, and 10 wt.% catalyst loading relative to the feedstocks. The mass/molar ratio of Fe/DM and AC in a dual catalyst was also varied. The yields of noncondensable gas, pyrolysis oil, and solid residue—which involve product distribution analyses according to ASTM D86 standards—into naphtha-like, kerosene-like, diesel-like, and LR fractions were obtained from both the actual experiment and theoretical calculations and are summarized in Table S2. The differences between the actual, experimental, and theoretical values are illustrated in Figure 5, revealing the synergy of the dual Fe/DM-AC catalyst system [38]. Notably, the temperature and residence time influence the thermal degradation reaction, and temperature plays a crucial role in thermal decomposition between 300 and 450 °C, which is consistent with the TGA/dTG analysis of PPW. Significant weight loss correlates with the cleavage of long hydrocarbon chains, resulting in the random scission of C-H and C-C bonds to form free radicals, leading to the randomized bond cleavage of large hydrocarbon chains into free radicals. These radicals then rearrange into shorter hydrocarbons while undergoing thermal degradation, and they induce β-scission of C-C bonds, producing shorter hydrocarbons, which can further decompose into even smaller hydrocarbons. After that, the AC catalyst has abundant acid active sites on the large pore surface structure, which increase the degree of hydrogen transfer, facilitating β-scission of the C-C bond to form a smaller hydrocarbon, which occurs on the AC pore structure surface, and the catalytic reaction of light hydrocarbon chains, e.g., hydrogenation, isomerization, oligomerization, and aromatization, into a naphtha-like fraction consisting of light aliphatic hydrocarbons. Notably, increased AC catalyst activity accelerated the conversion of long hydrocarbon chains into shorter chains through catalytic activity with acidic active sites and micropores, leading to pore-selective and secondary cracking reactions and further conversion into LR fractions and conversion of light hydrocarbons into light straight aliphatic hydrocarbons in the naphtha-like (C_5_–C_11_) fraction.

In contrast, the pyrolysis of PPW using the Fe/DM catalyst involves thermal decomposition, resulting in free radicals and smaller hydrocarbon radicals that continuously decompose into mid-volatile vapors, thereby accelerating the breakdown into straight-chain volatile vapors, which diffuse through a mesoporous structure on the DM catalyst to increase the catalytic activity and then facilitate hydrogen transfer at Fe–doped acidic active sites on the DM support template [18,21,26,27], which has a large mesoporous surface structure that further enhances catalytic cracking via hydrogenation, isomerization, and oligomerization, resulting in low-strength aliphatic hydrocarbons with the highest yield in the kerosene-like (C_11_–C_15_) range.

Figure 5 illustrates the trend of significant positive synergy observed in the kerosene-like fraction when the proportion of Fe/DM to AC catalyst increased to the Fe/DM-AC mass/molar ratio at 0.8:0.2, 0.6:0.4, 0.5:0.5, 0.4:0.6, and 0.2:0.8, yielding 11.11%, 21.85%, 18.88%, 27.27%, and 43.69% and declining to 15.78%, respectively, are used respectively. This trend can be attributed to enhanced thermal degradation, which involves the random scission of C-H and C-C bonds, forming free radicals and facilitating smaller volatile vapors through β-scission and yielding both H_2_ and small noncondensable gases. These volatile vapors can further crack into smaller hydrocarbons with acidic active sites and the micropore surface structure of the AC catalyst. Therefore, C-C bond cleavage also continues and occurs via hydrogen transfer on the surface of the AC catalyst. The increased acid strength from the AC catalyst promoted the β-scission of C-C bonds, resulting in smaller hydrocarbon chains. Furthermore, the pore distribution and large surface area of the AC catalyst facilitate hydrogen transfer and hydrogenation reactions of shortened hydrocarbon chains on the acid strength active sites, leading to pore-selective reactions, isomerization, and oligomerization of light hydrocarbon chains into a naphtha-like fraction consisting of light aliphatic hydrocarbons. Conversely, increasing the proportion of Fe/DM enhances the cracking of hydrocarbon radicals and free radicals from thermal cracking into smaller volatile vapors, accelerating hydrogen transfer on Fe-doped vapors [18,21,26,27], which illustrates that the acidic active sites and mesopore surface structure yield smaller volatile vapors that can diffuse into mesoporous structures, enabling hydrogenation, isomerization, and oligomerization of mid-volatile vapor that further enhances accelerated conversion to straight aliphatic hydrocarbon compounds in the C_11_–C_15_ range. The reduced proportion of the AC catalyst in the Fe/DM-AC mass malar ratio resulted in a decrease in the contribution of the microporous structure and strength acid active sites with a large surface structure, thus decreasing the catalytic activity, such as oligomerization and aromatization, which reduces the ability of the AC catalyst to convert into smaller straight aliphatic hydrocarbon compounds, which is also consistent with the trend of positive synergy in noncondensable gas yield as the mass/molar ratio of the AC catalyst increases due to the acid strength active sites of both the Fe/DM and AC catalysts, accelerating the cracking of mid-hydrocarbon radicals to undergo hydrogen transfer and hydrogenation reactions, yielding smaller volatile vapors that diffuse through micro- and mesopore surface structures, as well as isomerization, oligomerization, and aromatization, resulting in smaller volatile hydrocarbon compounds in the naphtha-like range before further cracking into noncondensable gases. Both the Fe/DM and AC catalysts effectively crack mid-volatile vapors into shorter volatile vapors, illustrating an increasing trend in the synergistic effect on noncondensable gas from 1.91%, 4.58%, 10.60% and declined to 5.37% when Fe/DM-AC mass/molar ratios of 0.8:0.2, 0.6:0.4, 0.5:0.5 and 0.4:0.6, respectively while Fe/DM-AC of 0.2:0.8 mass/molar ratio was used, exhibit neither positive synergy nor a negative synergy. This results in a positive synergy for noncondensable gases when the proportion of AC is increased to the Fe/DM mass/molar ratio, which can be developed into a dual catalyst that efficiently converts large hydrocarbon chains into smaller volatile vapors and catalyzes via hydrogenation, isomerization, and oligomerization, including further secondary cracking reactions to form shortened straight aliphatic hydrocarbon compounds in the C_9_–C_15_ range. In contrast, the naphtha-like fraction did not exhibit a significantly trend of negative or positive synergy effect when the proportion of Fe/DM to AC mass/molar ratio was increased to 0.8:0.2, 0.6:0.4, 0.5:0.5, 0.4:0.6, 0.2:0.8 yielding −20.57%, −33.73%, 1.40%, −4.71%, and −6.48%, respectively. The use of Fe/DM combined with the AC catalyst significantly affects the decrease in the naphtha-like fraction. This can be explained by the fact that thermal cracking significantly produces free radicals and hydrocarbon radicals via the β-scission of large hydrocarbon chains into mid-hydrocarbon radicals. The acidic active site and microporous structure of the AC catalyst promote hydrogen transfer and hydrogenation, resulting in the formation of straight hydrocarbon chains that continue to crack appropriately via strong acid activity and then diffuse into the micropore structure of the catalyst to further catalytic activity to produce straight aliphatic hydrocarbons. An increase in the ratio of the mass of Fe/DM to the mass of AC enhances the role of Fe/DM in hydrogen transfer at acid strength active sites on Fe_2_O_3_ supported on a DM support template that sufficiently diffuses into a mesoporous structure with a large surface structure, promotes the catalytic cracking of mid-hydrocarbon compounds, and then enhances accelerated catalytic cracking, hydrogenation, isomerization, and oligomerization, resulting in a decrease in the strength of aliphatic hydrocarbons with the highest yield in the C_11_–C_15_ carbon range [29,30]. However, the synergistic effect of the diesel-like and LR fractions obtained from the actual experiment, compared with the theoretical calculations, does not result in significant positive or negative synergy. Notably, the dual catalyst, with its high catalytic activity from acidic active sites and pore selectivity on a large surface structure and successful diffusion into micro- and mesopores, facilitates the conversion of mid-volatile vapors from the thermal decomposition of large hydrocarbon radicals during thermal cracking, resulting in a catalytic reaction within the pore structure of the dual catalyst. However, increasing the proportion of Fe/DM on the AC catalyst did not result in a significant positive synergistic effect. This suggests that catalytic cracking via a dual catalyst is efficient in converting long-chain hydrocarbons into mid- and shorter hydrocarbons in the carbon range of C_5_–C_15_, whereas the diesel-like and LR fractions are influenced by catalytic activity through hydrogenation, hydrocracking, isomerization, oligomerization, and secondary cracking reactions. This results in the conversion of LR and diesel-like fractions into shorter straight aliphatic hydrocarbons in the naphtha-like and kerosene-like fractions when the dual catalyst is used. Furthermore, the synergistic effect of the ratio of Fe/DM to AC, which was developed as a dual catalyst in catalytic pyrolysis, on their product distribution cannot fully explain the positive effect (promoter) or the negative effect (inhibitor) when different mass/molar ratios of the two catalysts are used. These synergistic effects are consistent with the variation in the mass/molar ratio of the Fe/DM-AC dual catalyst, which enhances the conversion of long hydrocarbon chains into a noncondensable gas and pyrolysis oil, resulting in significantly different product distributions depending on the mass/molar ratio of the catalyst. Therefore, kinetic and thermodynamic analyses of these dual catalysts in catalytic pyrolysis will be conducted in future studies.

### 2.4. CCD and RSM Optimization of the Experimental Results

On the basis of preliminary univariate experiments, an optimization study was conducted using a central composite design (CCD) within a response surface methodology (RSM) framework. This approach aimed to model and optimize the yields of pyrolysis oil and the kerosene-like fraction. The CCD efficiently integrates factorial, axial, and center points, enabling the capture of linear, interaction, and quadratic effects of multiple factors. This design significantly reduces the number of experiments required while allowing for the modeling of curvature, which is crucial for exploring complex process responses. The RSM subsequently fits a polynomial model to the experimental data, facilitating the prediction and optimization of responses as functions of the factors. In this study, optimization focused on using a dual Fe/DM-AC catalyst with a 0.6:0.4 mass/molar ratio. However, in this experiment, it is difficult to control the parameters in detail to the significant digits. Therefore, the operation conditions in each parameter were performed without decimals. The investigation determined the optimal process conditions by varying three factors according to the CCD: temperature (A), N_2_ flow rate (B), and catalyst loading (C). The second-order models were fitted to the experimental data for both pyrolysis oil and kerosene-like fraction yields. This well-established approach for chemical process optimization quantifies the effect of each factor and its interactions on the response, thereby identifying optimal conditions with a manageable number of experimental runs. The design parameters, levels, and resulting wt.% values for the desired pyrolysis yield and kerosene-like fraction are presented in Table 5. The experimental data were analyzed via regression analysis and analysis of variance (ANOVA) with two-parameter response variables.

Table 6 presents the ANOVA results for the pyrolysis oil yield. Conducted via a factorial experimental design and CCD, the ANOVA examined the sources of variability in the model at a statistical significance level of 0.05. The design, which included temperature as a factor, revealed that conditions with a *p* value (probability > F) less than 0.05 were statistically significant. Specifically, temperature critically influences the percentage yield of pyrolysis oil. The lack-of-fit test, with a *p* value (probability > F) less than 0.05, indicated that the regression equation applied to the data was appropriate with 95% confidence [23,24,25,41]. The results confirmed that the fitted quadratic model was statistically significant (F = 7.0709, *p* = 0.0026). A larger F value and a smaller *p* value correspond to a more significant term. Temperature (A) was the most dominant factor in the model, with the largest sum of squares (712.887) and the highest F value (44.2725), with *p* < 0.0001, indicating a highly statistically significant effect on pyrolysis oil yield. In contrast, the main effects of the N_2_ flow rate (B) and catalyst loading (C) were not statistically significant, with *p* values greater than 0.05 (approximately 0.0995 and 0.3348, respectively). This suggested that their individual linear contributions were minor within the tested range. Among the interaction terms, only the AB interaction was marginally significant (F = 3.8431, *p* = 0.0784), indicating a significant interaction effect between temperature and N_2_ flow rate. The other interactions (AC and BC) were far from significant. With respect to the quadratic terms, only the N_2_ flow quadratic term (B^2^) was significant (F = 9.4818, *p* = 0.0117), implying a nonlinear effect with respect to N_2_ flow rate. The quadratic terms for temperature and catalyst loading were not significant. Therefore, temperature was the most influential factor affecting pyrolysis oil yield, and the effect of the N_2_ flow rate exhibited a modest curvature. These findings align with the general RSM interpretation, where significant terms (*p* < 0.05) are retained for prediction, whereas nonsignificant terms may be omitted for model simplicity.

Table S3 summarizes the statistical evaluation of linear and quadratic models for predicting pyrolysis oil yield from the catalytic pyrolysis of packaging plastic waste (PPW). Both models demonstrated statistical significance, with *p* values (probability > F) less than 0.05, confirming that the relationships between the process variables and pyrolysis oil yield were adequately described. The lack-of-fit test results, shown in Table S4, with *p* values < 0.05, further support that both the linear and quadratic regressions reliably fit the experimental data with 95% confidence.

Model adequacy, determined via the R-squared (R^2^) values shown in Table S5, revealed that the quadratic model (R^2^ = 0.8642) explained 86.42% of the variability in pyrolysis oil yield, outperforming the linear model (R^2^ = 0.6599). However, the adjusted R^2^ (0.742) was substantially lower. Generally, a desirable model has an R^2^ close to 1, with adjusted R^2^ and predicted R^2^ values within approximately 0.2 of each other for good predictability. The large discrepancy observed here, particularly the negative predicted R^2^, suggested potential overfitting or that important variability in the data remained unexplained by the model. The adequate precision value was 10.25, indicating a strong signal-to-noise ratio in the model’s fit. The coefficient of variation (CV) was 5.73%, suggesting low experimental scatter relative to the mean response. These results justified the use of the quadratic CCD model for optimization while acknowledging its limitations in predicting new data. A significant lack-of-fit term (F ≈ 406.9, *p* < 0.0001) was also observed. Normally, a significant lack of fit suggests that the model does not fully capture the systematic variability, possibly due to of higher-order effects or noise not accounted for by the second-order model. This is plausible given the complex chemical mechanisms involved in pyrolysis reactions, including temperature and residence time effects that accelerate bond cleavage, leading to free radical formation, and the influence of catalytic activities such as hydrogen transfer, hydrogenation, hydrocracking, isomerization, oligomerization, and aromatization, as discussed in the univariate study section. The inherent complexity of thermochemical processes and factors such as the feedstock composition, catalytic activity, and heating rate under isothermal conditions can contribute to unexplained variability, which is common in pyrolysis studies.

**Figure 6:**
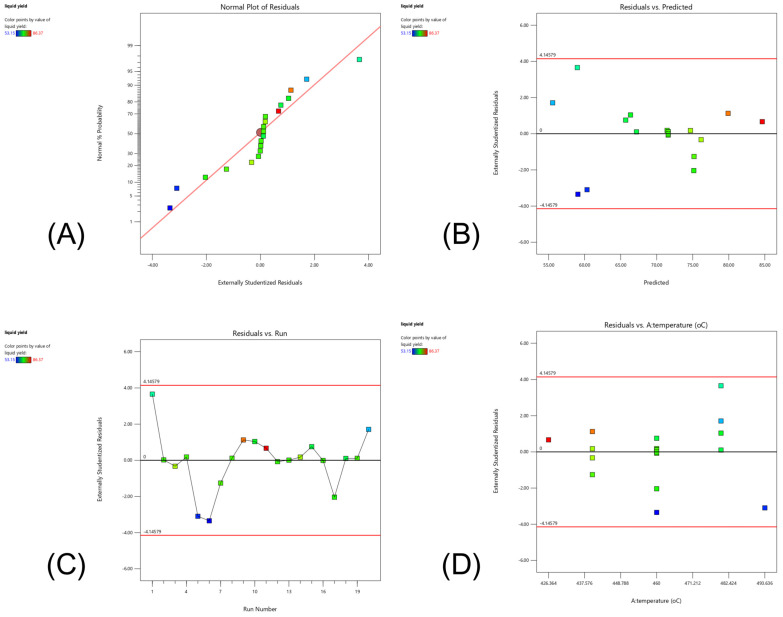
Analysis of residuals from the model of catalytic pyrolysis of plastic packaging waste on pyrolysis oil yield: (**A**) residual analysis; (**B**) residuals and predicted values; (**C**) predicted values and experimental values; (**D**) residuals and temperature parameters.

Figure 6A shows the normal probability plot of the externally studentized residuals, which assesses the normality of the data distribution. The plot shows that the residuals closely aligned with a straight line, confirming that the pyrolysis oil yield data followed a normal distribution. Figure 6B illustrates the homogeneity of variance, as shown by the plot of the externally studentized residuals versus the predicted values. This plot exhibited a random, symmetric distribution around zero, indicating constant variance across the dataset and demonstrating good model compatibility and reliability [42,43]. Figure 6C depicts the independence of data points by plotting externally studentized residuals against the run number. The absence of a specific pattern or trend indicated no systematic error, confirming an unbiased model with randomly scattered residuals and independent data points regarding the liquid product yield percentage. Figure 6D shows the effect of temperature on the residuals by plotting the externally studentized residuals against the temperature parameter. This plot showed no obvious violation of the assumption; the residuals were roughly normal and randomly scattered, supporting an unbiased fit. The even distribution of the liquid product yield percentage, both positively and negatively, indicated homogeneity of variance and data stability across varying temperature conditions. Overall, the residual analysis confirmed that the quadratic model was statistically robust, validating the assumptions of normality, homogeneity, and independence. These diagnostic tests, along with the high R^2^, supported its reliability in optimizing the liquid yield from the catalytic pyrolysis of PPW [42,43].

**Figure 7:**
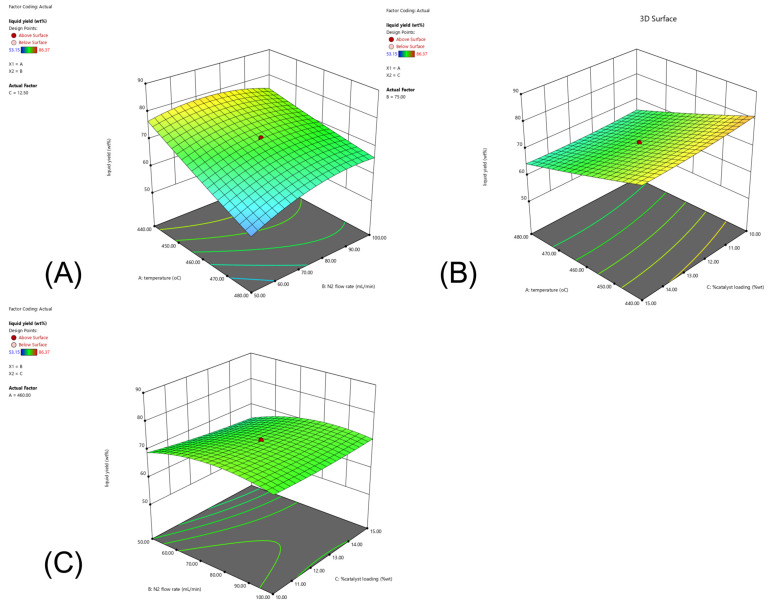
Three-dimensional surface response of the parameters of catalytic pyrolysis to pyrolysis oil yield: (**A**) temperature and nitrogen flow rate; (**B**) temperature and percentage of catalyst; (**C**) nitrogen flow rate and percentage of catalyst.

The statistical results confirmed that temperature was the primary factor influencing pyrolysis oil yield. Higher temperatures accelerated bond cleavage and cracking, increasing pyrolysis oil yields up to a certain point, which was consistent with the significant positive linear effect of temperature (A). The significant quadratic effect of N_2_ flow (B^2^) implied the existence of an optimum N_2_ flow rate. Too low a flow rate can lead to noncondensable gas build-up from longer residence times and increased secondary cracking reactions, whereas too high a flow rate may excessively entrain noncondensable gases, both of which reduce the pyrolysis oil yield. The nearly significant AB interaction term suggested that the conversion of PWW to pyrolysis oil was also affected. These curvature and interaction behaviors were visually evident in the 3D response surface plots, as shown in Figure 7, which illustrates a peak where the pyrolysis oil yield was maximized at a particular combination of temperature and N_2_ flow rate. The validated quadratic model surfaces demonstrated that the oil yield increased with temperature before levelling off, and a mild saddle shape was observed due to the AB interaction.

pyrolysis oil yield (wt.%) = 71.593 − 7.225A + 1.971B − 1.100C + 2.781AB + 0.451AC + 1.061BC + 0.318A^2^ − 3.255B^2^ + 0.602C^2^ (1)

pyrolysis oil yield (wt.%) = 650.937 − 1.622(temperature) − 1.911(N_2_ flow rate) − 8.275(% catalyst loading) + 0.006(temperature × N_2_ flow rate) + 0.009(temperature × % catalyst loading) + 0.017(N_2_ flow rate × % catalyst loading) + 0.001(temperature)^2^ − 0.005(N_2_ flow rate)^2^ + 0.096(% catalyst loading)^2^ (2)

For the kerosene-like fraction yield, the model selection criteria, detailed in Table S6, indicated that a two-factor interaction (2FI) model was the most appropriate. Both the linear and 2FI models demonstrated statistical significance (*p* < 0.05). However, sequential testing revealed that adding full quadratic terms did not significantly improve the model fit compared with the 2FI model (2FI vs. linear: F = 6.590, *p* = 0.0060; quadratic vs. 2FI: F = 0.628, *p* = 0.6135). Furthermore, the lack-of-fit test results presented in Table S7 supported the 2FI model as the most suitable mathematical representation. This conclusion was further reinforced by the higher R-squared value of the 2FI model, as shown in Table S8, which indicated a stronger fit and greater accuracy than the linear model in describing the relationships between the experimental factors and the kerosene-like yield. Therefore, the 2FI model was selected as the most appropriate model for this analysis.

Analysis of variance (ANOVA), presented in Table 7, was used to examine the sources of variation in the selected 2FI model at a statistical significance level of 0.05. The ANOVA confirmed that the overall model fit was significant (F = 15.574, *p* < 0.0001), indicating that the chosen terms explained a substantial portion of the total variance. Temperature (A) was the most influential factor, with a large sum of squares (SS = 35.123) and a highly significant F value (F = 67.49, *p* < 0.0001). Catalyst loading (C) also had a modest but statistically significant effect (F = 5.828, *p* = 0.0312). The main effect of the N_2_ flow rate (B) was not significant (F = 0.355, *p* = 0.5615).

A key finding was the highly significant interaction between temperature and the N_2_ flow rate (AB interaction: F = 18.77, *p* = 0.0008). This indicated a strong synergistic or antagonistic effect between temperature and the N_2_ flow rate on the kerosene-like yield, in contrast to the pyrolysis oil yield, where the AB interaction was only marginally significant. The other two-factor interactions (AC and BC) were not statistically significant. Among the quadratic terms included in the 2FI model (only B^2^ was included on the basis of model selection), B^2^ was significant (F = 17.90, *p* = 0.0028), suggesting a curved, nonlinear relationship between the N_2_ flow rate and kerosene-like yield. Overall, the kerosene-like fraction yield was driven primarily by temperature and the interaction between temperature and the N_2_ flow rate, with catalyst loading having a lesser, although still significant, influence. Higher temperatures and potentially higher catalyst loadings tend to promote further cracking of middle hydrocarbon chains into smaller hydrocarbons and noncondensable gases, whereas the dependence on the N_2_ flow rate is nonlinear. The lack-of-fit test (*p* value < 0.05) further supported the appropriateness of the regression model and its good fit to the data at the 95% confidence level.

The model fit exhibited an R^2^ of 0.8779 and an adjusted R^2^ of 0.8215, indicating that approximately 87.8% of the variability in kerosene-like yield was explained by the model and that a good proportion of the model terms were significant. The predicted R^2^ was 0.5386, which was lower than the adjusted R^2^ but still positive, suggesting some predictive capability. The difference between the adjusted and predicted R^2^ values (approximately 0.283) suggested moderate overfitting. The adequate precision value was 14.12, confirming an excellent signal-to-noise ratio in the model’s fit. The low coefficient of variation (CV) of 3.47% indicated very consistent experimental data. A significant lack of fit was also detected for this model (F = 15.57, *p* < 0.0001). While this statistically implies that the model does not capture all systematic variation, potentially because of unmodeled factors or complex kinetics inherent in pyrolysis reactions (e.g., feedstock heterogeneity), the high R^2^ and adequate precision values indicate that the model remains highly useful for understanding factor effects and for optimization purposes. Some residual variation is expected in complex thermochemical processes.

**Figure 8:**
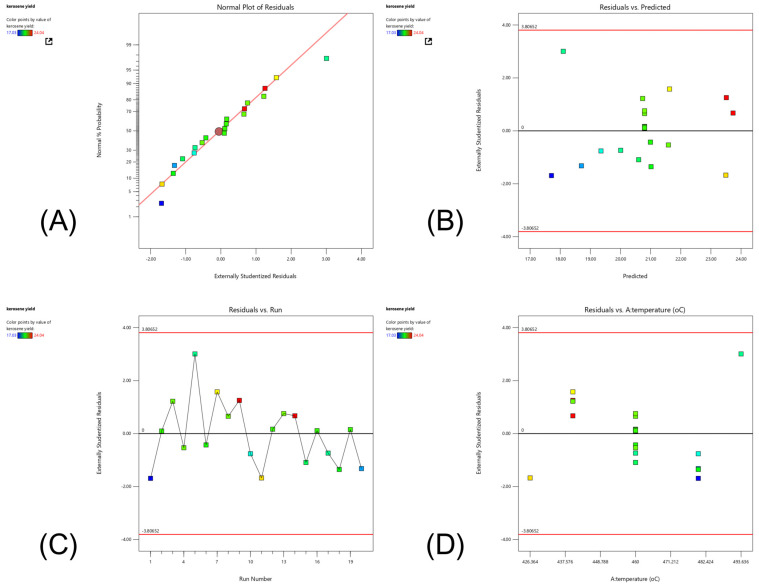
Analysis of residuals from the model of catalytic pyrolysis of plastic packaging waste on the kerosene-like fraction: (**A**) residual analysis; (**B**) residuals and predicted values; (**C**) predicted values and experimental values; (**D**) residuals and temperature parameters.

Diagnostic plots further supported the validity of the selected model. Figure 8A shows the normal percentage probability plot of the externally studentized residuals used to assess the normality of the data distribution. The residuals aligned closely with a linear trend line, confirming that the kerosene-like yield data were approximately normally distributed. Figure 8B illustrates the homogeneity of variance by plotting the externally studentized residuals against the predicted values. The even distribution of both positive and negative residuals demonstrated homogeneity of variance and indicated good model reliability. Figure 8C shows the independence of the kerosene-like yield data by plotting the externally studentized residuals against the run number. The residuals were randomly scattered with no specific pattern, showing a consistent distribution and suggesting that the data points were independent. Figure 8D shows the effects of temperature on the residuals by plotting the externally studentized residuals against the temperature parameter. The even distribution of residuals in both the positive and negative directions confirmed the homogeneity of variance under varying temperature conditions.

On the basis of these results, the ANOVA identified temperature as the single most dominant factor influencing the kerosene-like fraction yield. The significant AB interaction indicated that the effect of temperature was dependent on the N_2_ flow rate; for example, increasing temperature might lead to a greater increase in kerosene yield at low N_2_ flow rates than at high N_2_ flow rates. The significant quadratic term for the N_2_ flow rate (B^2^) further suggested an optimal flow rate for maximizing the kerosene-like fraction. Catalyst loading, while less influential than temperature, played a statistically significant role. This 2FI model effectively expresses the interaction between the two most significant parameters and provides prediction equations for both the coded and actual values, as shown in Equations (3) and (4), respectively.

kerosene-like yield (wt.%) = 20.798 − 1.604A − 0.116B + 0.471C + 1.105AB +   0.1925AC + 0.1675BC(3)

kerosene-like yield (wt.%) = 650.937 − 1622(temperature) − 1.911(N_2_ flow rate) −   8.275(% catalyst loading) +       0.006(temperature × N_2_ flow rate) +            0.009(temperature × % catalyst loading) +         0.017(N_2_ flow rate × % catalyst loading) (4)

**Figure 9:**
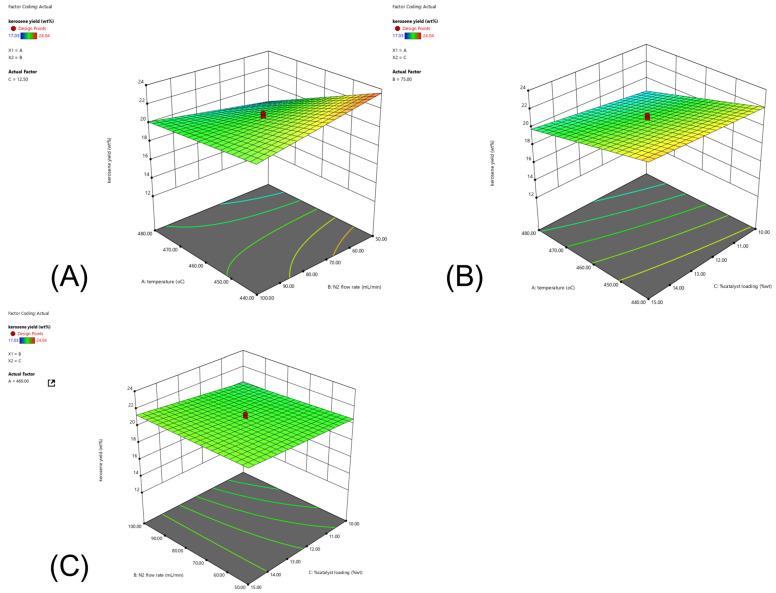
Three-dimensional surface response of the parameters of catalytic pyrolysis to the kerosene-like fraction: (**A**) temperature and nitrogen flow rate; (**B**) temperature and percentage of catalyst; (**C**) nitrogen flow rate and percentage of catalyst.

These curvature and interaction behaviors would be evident in the 3D surface response of the parameters of catalytic pyrolysis to the kerosene-like fraction as can be seen in Figure 9.

**Figure 10:**
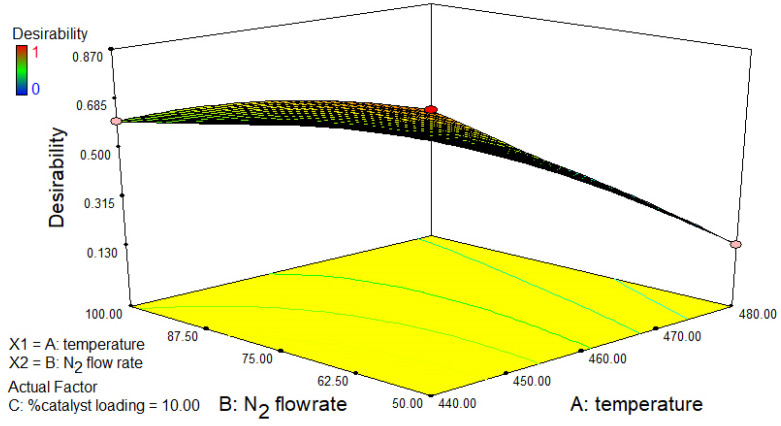
Three-dimensional surface response of the interaction of temperature and N_2_ flow rate of catalytic Pyrolysis to maximize both the yields of pyrolysis oil and kerosene-like fraction.

This study employed the use of CCD and RSM, which allowed simultaneous assessment of all factors and their interactions. The statistical analysis clearly shows that temperature is the most critical variable for both pyrolysis oil and kerosene-like fraction yields, with N_2_ flow rate (through its quadratic term) and catalyst loading having secondary effects. Model adequacy metrics (high R^2^, Adeq Precision > 4) confirm that the fitted models are statistically sound for describing the experimental data. The ANOVA (Table 6 and Table 7) and lack-of-fit tests (Tables S3–S7) guide which terms are significant and must be retained: for pyrolysis oil yield, only A and B^2^ (plus a weak AB) are important, whereas for kerosene-like fraction yield, A, C, AB, and B^2^ are key. Table S3 shows the quadratic model’s R^2^ = 0.8642 versus the linear model’s 0.6599, and Table S6 shows the significant improvement of the 2FI model for kerosene-like fraction yield (F = 6.590, *p* = 0.0060). Overall, CCD/RSM provided a rigorous framework for optimization: it identified the best-fit models and key variable effects, and it allows for the prediction of yields within the experimental domain. The high F values and low *p* values attest to the statistical significance of the models. While significant lack-of-fit in both cases indicates complex underlying chemistry and suggests that additional factors (or higher-order models) could be explored in future work, the current models capture the dominant behavior and enable identification of optimal pyrolysis conditions with confidence. Therefore, the optimization considered a temperature range of 426–493 °C, a N_2_ flow rate of 33–117 mL/min, and a catalyst loading of 8–17 wt.% relative to the feedstock weight. The optimization goal was to maximize both the pyrolysis oil yield and the yield of the kerosene-like fraction, as shown in Table S9. Under these conditions, design expert analysis identified the optimal conditions for maximizing the pyrolysis oil and kerosene-like yield as follows: a temperature of 440 °C, a N_2_ flow rate of 50 mL/min, and a combination of Fe/DM with the AC catalyst at a 0.6:0.4 mass/molar ratio, whereas the catalyst loading was 10 wt.%, the predicted yields were 79.91 wt.% for pyrolysis oil and 23.51 wt.% for the kerosene-like fraction as can be seen from Table S9 and 3D surface response of the interaction of temperature and N_2_ flow rate of catalytic pyrolysis to maximized both of the yielded of pyrolysis oil and kerosene-like fraction in Figure 10. Validation experiments conducted under these optimized conditions yielded 79.6 ± 0.35 wt.% pyrolysis oil and a 22.3 ± 0.22 wt.% kerosene-like fraction. These experimental results closely match the predicted values, with deviations of only 0.4% and 5.4% for the pyrolysis oil yield and kerosene-like fraction, respectively, demonstrating the reliability and accuracy of the model predictions.

### 2.5. Characterization of Pyrolysis Oil

#### 2.5.1. Chemical Compounds via GC/MS

The pyrolysis oil from catalytic pyrolysis under the optimum process conditions, which was obtained at 440 °C, a N_2_ flow rate of 50 mL/min, and a catalyst loading of 10 wt.% to feedstocks with a 0.6:0.4 Fe/DM-AC mass/molar ratio, was analyzed via gas chromatography–mass spectrometry. The chemical compounds at several retention times and the percentage relative peak areas (%) determined via gas chromatography–mass spectrometry (GC–MS) revealed the NIST chemical library, which properly identified the species and chemical compounds, and the formula weights consisting of C_5_–C_19_^+^ are listed in Table 8. The results of the GC/MS analyses could be divided into the carbon ranges of naphtha-like (C_5_–C_11_), kerosene-like (C_11_–C_15_), diesel-like (C_15_–C_19_ hydrocarbon compounds) and LR (C_19_^+^- hydrocarbon compounds) [44]. The results revealed that the relative percentage peak areas for noncatalytic pyrolysis resulted in naphtha-like, kerosene-like, diesel-like, and LR fractions of 34.322%, 21.377%, 13.982% and 30.319%, respectively. The use of the DM catalyst resulted in relative percentage peak areas of the naphtha-like, kerosene-like, diesel-like, and LR fractions of 37.375%, 35.575%, 17.979% and 9.071%, respectively. Notably, the use of Fe/DM in the catalytic pyrolysis of PPW resulted in relative percentage peak areas of the naphtha-like, kerosene-like, diesel-like, and LR fractions of 34.867%, 43.238%, 19.533% and 2.362%, respectively.

The influence of the DM and Fe/DM catalysts revealed that the acid strength active site, which is a pore-selective with a mesoporous surface structure, accelerated the thermal degradation of the shortened volatile vapor that diffused into the mesopore structure of the DM catalyst. The Fe/DM acidic active site promoted cracking at the active site and further cracking reactions, as well as hydrogenation, hydrocracking, isomerization, and oligomerization, resulting in the conversion of long-chain hydrocarbons to the LR fraction and further cracking to shorten hydrocarbon chains. An increase in the diesel-like fraction compared with that of the noncatalyst, DM, and Fe/DM resulted in a significant decreasing trend of LR conversion from 30.319%, 9.071%, and 2.362%, respectively. This also demonstrates the ability to use DM and Fe/DM catalysts, especially the presence of acidic active sites from the Fe-doped catalyst to the DM catalyst, which enhances the catalytic cracking ability. Additionally, the catalytic pyrolysis of PPW using an AC catalyst revealed the conversion of long hydrocarbon chains to the LR fraction (2.362%), diesel-like fraction (19.533%), kerosene-like fraction (43.238%), and naphtha-like fraction (34.867%). These results are consistent with the ability of the strong acid active site and micropores on the large surface structure to convert long-chain hydrocarbons to light hydrocarbon compounds in the kerosene-like and naphtha-like ranges at the highest proportion of 78.105%. Furthermore, the influence of the thermal decomposition of long-chain hydrocarbons initially induces the cleavage of C-C bonds and C–H bonds to form free radicals and hydrocarbon radicals, which then undergo β-scission reactions, resulting in the formation of shortened volatile vapors. The AC catalyst promotes C-C bond cleavage through hydrogen transfer and hydrogenation reactions. These reactions facilitate the reaction of shortened volatile vapors with acidic active sites, and the large microporous surface structure of the AC catalyst accelerates the isomerization and oligomerization reactions of intermediate volatile vapors on the porous surface, leading to the production of saturated and unsaturated hydrocarbons. Additionally, a relative percentage peak area of aromatic compounds was observed at 1.367%, resulting from aromatization reactions combined with isomerization and oligomerization that enhanced the successive cyclization of linear alkanes, resulting in the production of cyclic paraffins and cyclic olefins before undergoing a subsequent reaction through a secondary cracking reaction into light hydrocarbon compounds in the naphtha-like range of C_5_–C_11_ carbon atoms, revealing the catalytic performance of the activated carbon catalyst. The catalytic pyrolysis of PPW via the Fe/DM-AC dual catalyst presented a relative percentage area peak of LR and a diesel-like fraction of 18.593%, a kerosene-like fraction of 53.472% and a naphtha-like fraction of 27.935%, which included the role of the AC catalyst and Fe-doped DM catalyst, resulting in a positive synergistic effect from both the Fe-doped acid strength active site that promoted the hydrogen transfer reaction and further C-C bond cleavage through the catalytic activity of the Fe/DM and acid strength active site AC catalysts, accelerating the cracking of mid-hydrocarbon radicals to undergo hydrogen transfer and hydrogenation reactions, yielding smaller volatile vapors that diffuse through micro- and mesopore surface structures, as well as isomerization, oligomerization, and aromatization, resulting in smaller volatile hydrocarbon compounds in the kerosene-like and naphtha-like ranges before a smaller volatile vapor from the naphtha-like fraction may further crack into noncondensable gases. Both the Fe/DM and AC catalysts effectively cracked mid-volatile vapors into shorter volatile vapors.

#### 2.5.2. FT-IR Analyses

**Figure 11:**
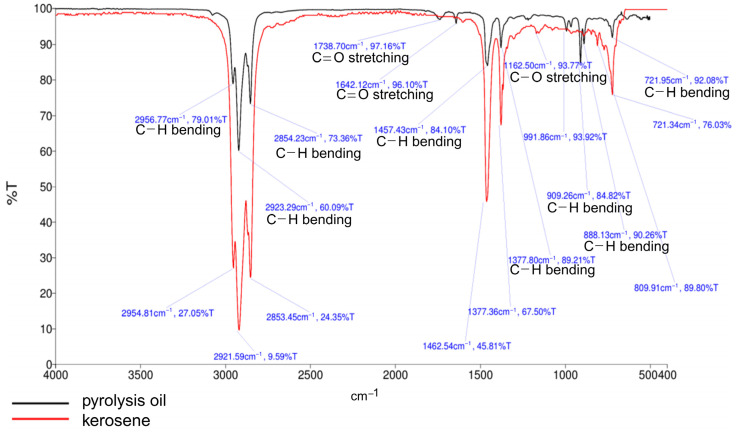
FT−IR analyses of pyrolysis oil.

The pyrolysis oil was analyzed via Fourier transform infrared (FT-IR) spectroscopy, as shown in Figure 11. The identified functional groups included the C-H bending aromatic group at 721.08 cm^−1^, the C-H bending aromatic hydrocarbon at 809.91 cm^−1^, the C-H bending of the alkene at 887.97 cm^−1^ and 965.41 cm^−1^, the C-O stretching at 1217.16 cm^−1^, the C-H bending (symmetric and asymmetric) of the methyl group in the alkane at 1457.48 cm^−1^, and the C-H stretching (symmetric and asymmetric) of the methylene group in the alkane at 2852.53 cm^−1^, 2921.68 cm^−1^, and 2956.57 cm^−1^. The results indicated the presence of alkane and alkene functional groups, suggesting the successive thermal cracking and catalytic cracking of hydrocarbon compounds, which are the primary components of LDPE in plastic packaging waste. The detection of carbonyl and ether groups suggests the presence of vinyl esters, resins, adhesives, or other chemical additives. These substances are commonly incorporated into plastic packaging to increase functionality, act as interlayer bonding agents, improve durability, or provide moisture resistance. The presence of these materials can be attributed to the polymers, plasticizers, or other ad hoc materials used in the manufacturing process.

#### 2.5.3. Physicochemical Analyses

Table 9 shows the results of physicochemical analyses of pyrolysis oil from the catalytic pyrolysis of PPW under optimal conditions. At 440 °C, a N_2_ flow rate of 50 mL/min, a catalyst loading to feedstock of 10 wt.%, and a mass/molar ratio of Fe/DM to AC catalyst of 0.6:0.4 were also determined. Notably, the pyrolysis oil from thermal decomposition represented elemental analysis consisting of only carbon (84.43%) and hydrogen (15.57%) resulting in an H/C ratio of 2.21, resulting in an HHV of 41.27 MJ/kg because the influence of thermal cracking accelerated the randomized C–C and C–H bond cleavage to form free radicals and hydrocarbon radicals. These radicals are further influenced by the hydrogen transfer reaction and β-scission of mid-hydrocarbon radicals and are continually degraded through secondary cracking reactions into smaller, volatile vapors, which may undergo repolymerization into large hydrocarbons and continue cracking into noncondensable gases. Additionally, kinematic viscosity analysis revealed a value of 36.14 mm^2^/s.

The elemental analysis revealed that the C, H, and H/C ratios did not significantly differ among the AC, Fe/DM, and Fe/DM-AC catalysts, as shown in Table 9, which explained that thermal cracking significantly produces free radicals and hydrocarbon radicals via β-scission of large hydrocarbon chains into mid-hydrocarbon radicals. The acidic active site and micro/mesoporous structure of the catalyst promote hydrogen transfer and hydrogenation, especially when the Fe-doped catalyst is combined with the DM catalyst to the AC catalyst, which illustrates the strong acid active site and large micro- and meso-porous surface structure, which strongly increase the catalytic activity and accelerate bond cleavage, resulting in the formation of straight hydrocarbon chains that continue to crack appropriately via strong acid activity and then diffuse into the micropore structure of the catalyst to further the catalytic activity of hydrogenation, isomerization, oligomerization and aromatization to produce straight aliphatic hydrocarbons and olefinic hydrocarbons. The physicochemical analysis revealed kinematic viscosities of 6.37, 6.69, and 4.31 mm^2^/s when AC, Fe/DM, and Fe/DM-AC, respectively, were used for catalytic pyrolysis. Furthermore, the higher heating value did not differ significantly when different catalysts were used, with the AC catalyst resulting in an HHV of 42.84 MJ/kg and the Fe/DM catalyst resulting in an HHV of 44.73 MJ/kg. However, the use of Fe/DM-AC presented an HHV of 44.92 MJ/kg.

## 3. Materials and Methods

### 3.1. Feedstock

**Figure 12:**
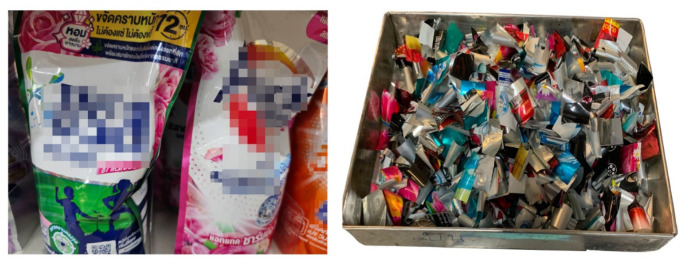
Laundry powder packaging is used as a sample for packaging plastic waste.

The packaging plastic waste (PPW) as a feedstock for catalytic pyrolysis was investigated. The material, consisting of used laundry powder packaging (Figure 12), was sourced from a supplier in Bangkok, Thailand. The thermal decomposition characteristic of PPW was analyzed using thermogravimetric analysis/differential thermogravimetry (TGA/DTG) on a Pyris Diamond instrument (PerkinElmer, USA). For each measurement, a 10 mg sample was placed in an alumina crucible and heated from 40 to 800 °C at a heating rate of 10 °C/min under a high-purity nitrogen atmosphere flowing at 50 mL/min. Prior to analysis, the system was purged with nitrogen (30 mL/min) at 150 K for 1 h. The temperature and sample weight loss were recorded throughout the experiment. The analysis was repeated three times to ensure the repeatability of the findings, as presented in Figure 1 and Figures S1 and S2.

Proximate and ultimate analyses of the PPW sample (approximately 2.5 × 2.5 cm) were conducted according to ASTM D 7582-10. The elemental composition (carbon, hydrogen, nitrogen, and sulfur) was determined via a LECO CHN-628 analyzer (LECO Corp., USA), and the difference in the oxygen content was calculated. The calorific values were measured via a LECO AC-500 bomb calorimeter (LECO Corp., St. Joseph, MI, USA) following ASTM D240. Additionally, the structures and chemical bonds of the hydrocarbon compounds in the PPW samples were analyzed via a Perkin Elmer Spectrum 100 Fourier transform infrared spectrometer (PerkinElmer, USA) with a dried KBr pellet. The absorbance was measured over a wavelength range of 4000–400 cm^−1^. The resulting spectra were similar to those of high-density polyethylene, low-density polyethylene, polypropylene, steripur, and polyester (Figures S3 and S4), with search scores of 0.989214, 0.988013, 0.9871148, 0.9897, and 0.970468, respectively, as shown in Figures S3 and S4.

### 3.2. Catalyst Preparation and Characterization

This study employed two types of catalysts: activated carbon (AC) and ferric oxide-impregnated dolomite (Fe/DM). Activated carbon (AC) was obtained from the Centre of Fuels Research and Energy from Biomass at Chulalongkorn University, Thailand. To prepare the biochar-activated carbon, waste empty palm kernels were carbonized at 350–400 °C for 3 h. The carbonized material underwent both physical and chemical activation, which involved exposure to heat steam at 900 °C for 3 h, and was carried out by adding KOH to the biochar at a 1:3 ratio and heating the mixture at 850 °C for 3 h.

The incorporation of ferric metal into a calcined dolomite (DM) framework is an effective strategy for increasing the acidity of the material. Dolomite, obtained from Chulalongkorn University, Thailand, was used as a support for ferric oxide. The preparation involved the calcination of dolomite in a muffle furnace at 800 °C for 3 h at a heating rate of 10 °C/min. Ferric oxide was incorporated into the calcined dolomite framework via wet impregnation, and this process generated Lewis and Brønsted acid sites, which are crucial for acid-catalyzed reactions [18,19,26]. An iron catalyst was subsequently prepared on dolomite via the wet impregnation method to study the catalytic efficiency and acid properties of iron as an active site. This involved preparing a solution of Fe(NO_3_)_3_·9H_2_O (Sigma Aldrich, Singapore) with mass loadings of 1 wt.%, 5 wt.%, and 10 wt.% relative to dolomite (approximately 5 g). Distilled water was added to dissolve the Fe(NO_3_)_3_·9H_2_O, and a water to Fe(NO_3_)_3_·9H_2_O ratio of 1.5 mL/g was used. The solution was stirred until complete dissolution and then slowly added dropwise to the dolomite framework until saturation. Afterward, the mixture was washed with deionized water and filtered through a vacuum filter. After drying at 105 °C for 3 h and then overnight at 120 °C, the obtained x%Fe/DM was calcined at 800 °C for 90 min at a heating rate of 10 °C/min under inert N_2_ gas.

X-ray diffraction patterns were obtained via a D8 Advance diffractometer (Bruker, USA) with Cu Kα (λ = 1.5406 Å) radiation operating at 40 kV and 40 mA. The scattering angle ranged from 5° ≤ 2θ ≤ 60°, and the scanning rate was 4°/min. Elemental analysis and chemical characterization of the catalyst surface were performed via an EDX 720 energy dispersive X-ray spectrometer (Shimadzu, Kyoto, Japan) and an S8 TIGER X-ray fluorescence spectrometer (Bruker Corp., Bremen, Germany). The morphological characteristics were analyzed via Brunauer–Emmett–Teller (BET) nitrogen adsorption–desorption analysis at 77 K via an ASAP 2020 instrument (Micromeritics Instrument Corp., Norcross, GA, USA). Prior to BET analysis, the catalyst samples were dried overnight at 105 °C and degassed at 573.15 °C for 6 h. The total pore volume, micropore pore volume, and specific surface area were calculated from the absorbed volume of nitrogen at a relative pressure (P/P_0_) of 0.99.

### 3.3. Experimental Design

In this study, an experimental design for optimization was employed via central composite design (CCD), which is a particular method in response surface methodology (RSM), to evaluate the effectiveness and sustainability of the method and provide more experimental information for the same experiment [41,42]. Therefore, the CCD and RSM were applied to determine the influence parameters; the values of the independent variables were equidistant from the center point, and the experiments were repeated at the center point. The independent variables are assigned three levels: −1, 0, and +1. The total number of experimental runs is 2^N^ (where N is the number of independent variables) plus an additional 4 runs at specific locations. These additional runs, referred to as axial points, are located at (+α, 0), (−α, 0), (0, +α), and (0, −α), where α (alpha) equals 2^N/4^. The design also includes experimental runs at the center point (0, 0). However, a CCD must carefully consider independent variables such as operating conditions that may not be suitable for the experiment or a negative value, which requires setting the range of independent variables, including the designated high and low levels of the independent variables, to be appropriate, and it is necessary to consider the α value properly according to the number of independent variables used in a CCD for reliability. Therefore, CCD was used to divide the three numerical parameters of temperature, N_2_ flow rate, and % catalyst loading into three levels, which were optimized via Stat-Ease Design Expert Version 7.0.0 (Stat-Ease Inc., Minneapolis, MN, USA) software; the correspondence between the actual and code values is shown in Table 10.

In this study, the CCD experimental design consisted of 8 factorial points, 6 axial points, and 6 replicates at the center point, comprising a total of 20 experiments to obtain the average data via Stat-Ease Design Expert via Equation (5).

(5) $$N=2^{n}+2n+n_{c}$$

where *N* is the total number of required experiments.

*n* is the number of parameters

*n_c_* is the number of replicates at the center point

To optimize the parameters influencing the pyrolysis reaction, a second-order polynomial model was fitted to the data obtained from each experiment via Equation (6):

(6) $$Y=\beta_{0}+\sum_{i=1}^{n}\beta_{i}X_{i}+\sum_{i=1}^{n}\beta_{ii}X_{i}^{2}+\sum_{i}^{n}\sum_{j>1}^{n}\beta_{ij}X_{i}X_{j}$$

where *Y* is the predicted response.

*n* is the total number of experiments

*β* represents the regression coefficient of the constant

*β_i_* where *i* represents the regression coefficient of the constant

*β_j_* represents the regression coefficient of the constant

*β_ij_* represents the regression coefficient of the constant

*x_i_* and *x*_0_ are coded independent parameters

Both graphical representations are numerically investigated by comparing the predicted values of the model with the experimental values, and the highest degree of fit of the model is obtained. The central composite design (CCD) facilitates the selection of an appropriate prediction model that relies on analysis of variance (ANOVA), which requires the following: (1) a normal distribution; (2) variance stability; (3) independence of residuals. In particular, the normal distribution of residuals is analyzed via an F test that accurately compares the variance between two data groups when the residuals are normally distributed. Furthermore, the residuals should have a similar range for both positive and negative values, indicating stable variance within the data, and the randomly scattered residuals, with no clear trends or patterns, suggest that the data points are independent of each other. This randomness indicates that the data were collected through random sampling, eliminating bias and ensuring that the independent variables can fully influence the dependent variable. The analyses can confidently use the two data groups to create a model and test the statistical significance of the independent variables. Key statistical values, such as the sequential *p* value, lack of fit, *p* value, R-square (R^2^), coefficient of variation (CV), adequate precision (AP), and predicted residual error sum of squares (PRESS), are then evaluated. Finally, the CCD can proceed with optimization—finding the optimal point under the constraints of the two independent variables then, satisfaction tests are conducted, and a response surface graph illustrates the relationship between the two independent variables effectively and the desirable level of the dependent variable via response surface methodology (RSM), which involves creating a three-dimensional response surface graph that illustrates the relationship between two independent variables and their effect on a dependent variable. It visually represents the relationship between these variables and allows for efficient optimization for the selection of optimal points under specific constraints. Additionally, the RSM aids in decision-making processes by considering both its optimal point and the desirable level for the experiments.

### 3.4. Experimental Procedure

**Figure 13:**
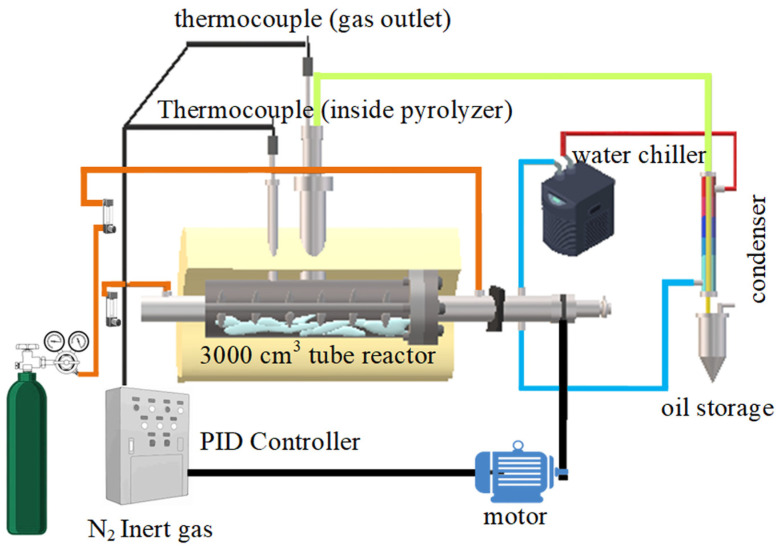
Schematic diagram of a 3000 cm^3^ custom-built pyrolysis reactor.

In this study, both the thermal degradation and catalytic reactions of PPW were carried out in a 3000 cm^3^ custom-built stainless-steel reactor (30 cm internal diameter × 60 cm reactor length) coupled with a condensing unit using a cool-water condensing system to condense volatile vapor into a liquid fraction under atmospheric pressure. Figure 13 shows a schematic diagram of the pyrolysis of PPW into kerosene-like liquid products. Prior to the experiment, the catalyst was added to the reactor at a load of 8.3–16.7 wt.% of the PPW weight, and approximately 250 g of PPW was packed into the reactor. Inert nitrogen gas (33.0–117.0 mL/min) was fed into the reactor, ensuring an oxygen-free atmosphere for the pyrolysis experiment. A constant flow rate of 50 mL/min N_2_ gas was used as the carrier gas in the pyrolysis reactions. A proportional–integral–derivative (PID) controller was heated to the desired temperature at a heating rate of 10 °C/min, and the reactor was heated to the desired temperature (426.4–493.6 °C). When the temperature inside the reactor reached 300 °C, the agitator was started. The reactor was agitated by a stainless-steel agitator at a constant rate of 30 rpm to ensure uniform heat and mass transfer through both the feedstock and the catalyst.

PPW undergoes thermal decomposition and carbon–carbon bond cleavage via a free-radical reaction, bond scission, and catalytic activity, producing small volatile vapors that pass through a cooling unit. The volatile vapor was condensed from the entrained pyrolysis oil. The first drop of pyrolysis oil was observed when the gas outlet temperature reached approximately 70 °C. The pyrolysis oil was then collected in the storage tank every 5 min until it was confirmed that the last drop of condensable volatile vapor had condensed, suggesting the completion of the reaction. The pyrolysis oil was collected in an oil storage tank, while uncondensed volatile vapors passed through the condensing unit, the gas drier, and noncondensable gases were released. The solid residue (including both solid residues, spent catalyst, and carbonaceous) was collected and weighed at room temperature. The liquid pyrolysis oil and solid yield were quantified by weighing, expressed as a percentage of their weight, while the gaseous products were calculated according to the mass balance. 

### 3.5. Product Yield and Composition Analysis

The mass balance of the catalytic pyrolysis of packaging plastic waste was determined by weighing the liquid condensate (*M*_2_). The total solid residue (*M*_4_), which included the pyrolysis solid residue, spent catalyst, and carbonaceous material, was obtained and weighed after the pyrolyzer had cooled to room temperature. The weight of the solid yield was calculated as the difference between the total solid residue and the initial weight of the catalyst used in the pyrolyzer (*M*_3_). The yield of liquid pyrolysis oil was calculated as the mass of the collected liquid condensate (*M*_2_) divided by the initial reactant mass. The yield of solid residues was determined on the basis of the mass change in the total solid and fresh catalysts divided by the initial reactant mass. The yield of gas was also calculated via mass balance owing to the difference between the initial reactant mass and the total mass of the liquid and solid products.

Therefore, the yield of each product was calculated via Equations (7) and (9):

(7) $$\%Y_{\mathrm{liquid}}=\frac{M_{2}}{M_{1}}\times 100$$

(8) $$\%Y_{\mathrm{solid}}=\frac{M_{4}-M_{3}}{M_{1}}\times 100$$

(9) $$\%Y_{\mathrm{gas}}=100-\left(Y_{\mathrm{liquid}}+Y_{\mathrm{solid}}\right)$$

To analyze the composition of the condensable pyrolysis oil and determine the optimal conditions, simulated distillation gas chromatography (DGC) was used to characterize the pyrolysis oil according to ASTM D86, which was carried out via an Agilent GC7890A gas chromatograph (Agilent Technologies, Palo Alto, CA, USA) coupled with a DB-1 capillary column (2.65 µm thickness, 100% dimethylpolysiloxane, 0.53 mm inner diameter, 10 m length) from J&W Scientific, Folsom, CA, USA. The pyrolysis oil fractions were classified on the basis of their boiling temperature range, which was determined according to the crude oil boiling temperature range [44]; a naphthalene-like fraction (C_5_–C_11_) was identified at the boiling point up to 200 °C, and a kerosene-like fraction (C_11_–C_15_) was obtained at the boiling point between 200 °C and 250 °C. The light gas oil fraction contained boiling points ranging from 250 °C to 300 °C, and the gas oil fraction also contained boiling points ranging from 300 °C to 370 °C. Therefore, a boiling point between 250 °C and 370 °C is known as the diesel-like fraction (C_15_–C_19_ hydrocarbons). The long residue (>C_19_) is defined by a boiling point exceeding 370 °C.

The GC/MS analysis was conducted via an Agilent GC7890/GCMS5978 gas chromatograph with a mass spectrometer (Agilent Technologies, Palo Alto, CA, USA) equipped with an HP-5 MS capillary column (0.25 µm thickness, 5%-phenyl)-methyl-polysiloxane, 0.25 mm inner diameter, 30 m length). High-purity helium (99.99%) was used as the carrier gas at a constant flow rate of 1 mL/min. The injector temperature was set to 250 °C. The column temperature was initially set at 40 °C and then increased to 250 °C at a rate of 3 °C/min. The mass spectrometer ion source temperature was maintained at 230 °C with an electron energy of 70 eV. Data analysis and the chemical compounds were identified by retention time and relative molecular weight on the basis of peak matching of the mass spectra against the NIST library.

Additionally, elemental analysis of the pyrolysis oil products was conducted via a LECO CHN-628 analyzer (LECO Corp., St. Joseph, MI, USA) to determine the carbon, hydrogen, and nitrogen contents. The oxygen content in the samples was obtained via different calculations from the elemental analysis. The physicochemical properties of the pyrolyzed oil from PPW were also examined. The kinematic viscosity was measured at 40 °C via a REOLOGICA Instruments AB viscoanalyzer (Rheometric Scientific Inc., New Castle, DE, USA) following the procedure of ASTM D445. The caloric value was measured via a bomb calorimeter following ASTM D240.

### 3.6. Synergy of Dual Catalytic Pyrolysis

To evaluate the synergistic interaction between the dual catalysts, a synergistic study was conducted using two different catalyst mass/molar ratios. The pyrolysis oil yield and kerosene-like yield were experimentally determined through catalytic pyrolysis according to the optimal conditions by varying the proportions of AC and Fe/DM. This variation emphasized both the pyrolysis oil yield and the kerosene-like fraction, which, compared with the theoretical value derived from the additivity rule from the proportional yield average, affected the product yield. According to this rule, the total yield is the sum of the individual contributions of each catalyst, and a linear relationship is assumed [38,39]. This approach may result in a positive or negative effect compared with that achieved using each catalyst alone. For each product distribution, the yield percentage (*Y_exp_*) was experimentally determined through the combination of AC and Fe/DM. The theoretical values (*Y_theo_*) were calculated via Equation (10):

(10) $$Y_{theo}=Y_{1}M_{1}+Y_{2}M_{2}$$

where *Y*_1_ and *Y*_2_ represent the actual product distribution yield percentages obtained from the catalytic pyrolysis of WPP using individual AC and individual Fe/DM, respectively. *M*_1_ and *M*_2_ represent the mass/molar ratios of AC and Fe/DM, respectively, in the additional catalyst combination. Additionally, *Y_Exp_* represents the experimental value from a dual catalytic test of the blends. The deviation value (Δ*Y*) was used to calculate the synergistic effect during the blending of AC and Fe/DM on the catalytic pyrolysis of PPW, as shown in Equation (11):

(11) $$\Delta Y=Y_{exp}-Y_{theo}$$

The value of Δ*Y* represents the strength of the synergistic reaction [40]. As shown in Equation (11), if the experimental value is greater than the theoretical value, blending with other catalysts enhances the positive effect on the product yield during the catalytic pyrolysis reaction, indicating that the combined catalysts increase the yield beyond the sum of their individual contributions. When the deviation value is equal to zero, there is no significant synergistic effect; the combined catalysts perform additively. There is no interaction of the dual catalyst when the deviation value < 0. A negative synergistic effect or inhibitory effect is observed, suggesting that the catalysts might hinder each other’s activity, which leads to a lower-than-expected yield. Therefore, this study aimed to understand whether and how the combined use of AC and Fe/DM catalysts could improve the efficiency of dual catalytic pyrolysis to produce higher yields of valuable pyrolysis oil, which also suggests a beneficial catalyst reaction chemical pathway.

## 4. Conclusions

This study investigated the catalytic pyrolysis of packaging plastic waste (PPW) to produce kerosene-like fractions via a systematic experimental approach. A factorial design with central composite design (CCD) and response surface methodology (RSM) was employed to optimize the process conditions for maximizing kerosene-like fractions and to elucidate the synergistic effects between activated carbon (AC) and a bifunctional acid catalyst (5%Fe/DM). The optimal operating conditions were determined to be 440 °C, a N_2_ flow rate of 50 mL/min, and 10 wt.% catalyst loading when a 0.6:0.4 mass/molar ratio of 5 wt.% Fe-doped DM catalyst combined with the AC catalyst was used. This optimal condition yielded a maximum pyrolysis oil content of 79.6 ± 0.35 wt.% and a kerosene-like fraction of 22.3 ± 0.22 wt.%. The experimental results closely aligned with the predicted values, indicating that pyrolysis oil yielded only a 2.8% deviation and the kerosene-like fraction yielded a 2.0% deviation. Further investigation into the synergistic effects of the Fe/DM and AC catalysts in the catalytic pyrolysis of PPW to kerosene-like fractions revealed a positive synergistic effect under optimal conditions when the mass/molar ratio was 0.6:0.4 (Fe/DM:AC). Increasing the mass ratio of the Fe/DM to AC catalyst further enhanced the positive synergistic effect, significantly promoting the cracking of PPW into light hydrocarbons. This enhanced cracking is attributed to thermal decomposition, which induced random bond cleavage of C–C and C–H bonds to form hydrocarbon radicals. The bifunctional catalyst, with its acid-strength active site of Fe on the DM template, facilitates catalytic cracking and hydrogen transfer reactions to produce smaller volatile hydrocarbon vapors and further cracks that can readily diffuse through the mesopore/micropore structure of the high surface area of AC, resulting in a hydrocarbon arrangement from the textural properties of AC. This, in turn, results in the selective formation of the C_5_–C_15_ hydrocarbon range.

## Figures and Tables

**Table 1:** Proximate and ultimate analyses.

Components	PPW	PE	PP	Standard Method
proximate analysis (wt.%)				ASTM D7582
volatile	90.28	96.13	97.24	
fixed carbon	2.89	3.87	2.76	
ash	6.83	n.d.	n.d.	
ultimate analysis (wt.%)				ASTM D7583
C	83.35	83.24	84.11	
H	14.73	16.76	15.89	
O ^a^	1.92	n.d.	n.d.	
N	n.d. ^b^	n.d.	n.d.	
S	n.d.	n.d.	n.d.	
H/C (mol/mol)	2.12	2.42	2.27	calculation
O/C (mol/mol)	0.02	n.a. ^c^	n.a.	calculation
HHV	39.08	42.85	41.82	ASTM D5865

^a^ by the difference calculation, ^b^ n.d. = not detected, ^c^ n.a. = not applicable.

**Table 2:** X-ray fluorescence analyses.

Element	Compound	Concentration (wt.%)			
		AC	DM	5% Fe/DM	10% Fe/DM
Al	Al_2_O_3_	n.d.	0.19	0.22	0.18
Si	SiO_2_	0.08	0.39	0.67	0.55
Ti	TiO_2_	n.d.	0.06	0.06	n.d.
Na	Na_2_O	0.42	0.33	0.41	0.83
Mg	MgO	0.16	33.01	31.82	31.72
P	P_2_O_5_	0.02	0.02	0.02	n.d.
K	K_2_O	0.54	n.d. ^a^	n.d.	n.d.
Ca	CaO	0.08	59.82	54.85	53.19
S	SO_3_	0.03	0.06	0.11	0.11
Fe	Fe_2_O_3_	0.01	0.23	5.82	9.14
other	Other	1.91	5.89	6.02	4.28

^a^ n.d. = not detected.

**Table 3:** EDX analyses of the oxide that formed after Fe was incorporated into the dolomite template.

Element	Oxide	Concentration of Fe-Doped (wt.%)		
		DM	5%Fe/DM	10%Fe/DM
Fe	Fe_2_O_3_	0.04	4.77	7.82

**Table 4:** Textural properties of the catalysts.

Catalyst	BET Surface Area (m^2^/g)	Total Pore Volume (cm^3^/g)	Total Pore Size (nm)
AC	830.77	0.05	2.37
calcined DM	19.95	0.10	18.79
5 wt.%Fe/DM	18.77	0.10	19.46
10 wt.%Fe/DM	17.03	0.10	25.80

**Table 5:** CCD parameters and responses.

Std	Variables			Product Distribution (wt.%)	
	Temperature (°C)	N_2_ Flowing Rate (mL/min)	% Catalyst Loading (wt.%)	Liquid Yield	Kerosene Fraction
1	440	50	10	82.47	24.04
2	480	50	10	64.63	17.03
3	440	100	10	75.37	21.25
4	480	100	10	68.76	19.02
5	440	50	15	75.11	24.03
6	480	50	15	59.18	18.15
7	440	100	15	72.36	22.27
8	480	100	15	67.45	20.45
9	426	75	12	86.37	22.52
10	493	75	12	54.63	19.57
11	460	32	12	53.15	20.72
12	460	117	12	67.64	19.93
13	460	75	8	70.68	19.54
14	460	75	16	71.93	21.25
15	460	75	12	71.31	20.92
16	460	75	12	72.04	21.27
17	460	75	12	71.63	21.34
18	460	75	12	71.68	20.87
19	460	75	12	72.02	20.91
20	460	75	12	71.56	20.88

**Table 6:** Analysis of variance (ANOVA) of the pyrolysis oil yield.

Source	Sum of Squares	df	Mean Square	F Value	*p* Value Prob > F	
Model	1024.711	9	113.857	7.0709	0.0026	significant
A-temperature	712.887	1	712.887	44.2725	<0.0001	
B-N_2_ flow rate	53.061	1	53.061	3.2952	0.0995	
C-%catalyst loading	16.536	1	16.536	1.0270	0.3348	
AB	61.883	1	61.883	3.8431	0.0784	
AC	1.629	1	1.629	0.1012	0.7570	
BC	9.010	1	9.010	0.5596	0.4717	
A^2^	1.455	1	1.455	0.0904	0.7699	
B^2^	152.678	1	152.678	9.4818	0.0117	
C^2^	5.229	1	5.229	0.3248	0.5813	
Residual	161.022	10	16.102			
Lack of Fit	160.628	5	32.126	406.9271	<0.0001	significant
Pure Error	0.395	5	0.079			
Cor Total	1185.733	19				
Std. Dev.	4.013	R-Squared		0.864		
Mean	69.999	Adj R-Squared		0.742		
C.V.%	5.733	Pred R-Squared		−0.028		
PRESS	1218.850	Adeq Precision		10.246		

**Table 7:** Analysis of variance (ANOVA) of the kerosene-like fraction.

Source	Sum of Squares	df	Mean Square	F Value	*p* Value Prob > F	
Model	48.630	6	8.105	15.574	<0.0001	significant
A-temperature	35.123	1	35.123	67.489	<0.0001	
B-N_2_ flow rate	0.185	1	0.185	0.355	0.5615	
C-%catalyst loading	3.033	1	3.033	5.828	0.0312	
AB	9.768	1	9.768	18.770	0.0008	
AC	0.296	1	0.296	0.570	0.4639	
BC	0.224	1	0.224	0.431	0.5228	
A^2^	6.766	13	0.520			
B^2^	6.537	8	0.817	17.898	0.0028	significant
C^2^	0.228	5	0.046			
Residual	55.395	19				
Lack of Fit	48.630	6	8.105	15.574	<0.0001	significant
Pure Error	35.123	1	35.123	67.489	<0.0001	
Cor Total	0.185	1	0.185	0.355	0.5615	
Std. Dev.	0.721	R-Squared		0.8779		
Mean	20.798	Adj R-Squared		0.8215		
C.V.%	3.469	Pred R-Squared		0.5386		
PRESS	25.557	Adeq Precision		14.1168		

**Table 8:** GC/MS analyses.

R.T (min)	A Relative Percentage Area Peak (%)					Chemical Compounds	Molecular Formular
	Non Cat	DM	Fe/DM	AC	Fe/DM-AC		
Straight aliphatic hydrocarbon compounds							
1.576	1.444	1.993	1.303	2.887		Pentane	C_5_H_12_
1.945	1.048					n-Hexane	C_6_H_14_
4.267	1.016	1.741	1.611	1.465	1.935	Octane	C_8_H_18_
6.029	1.014	1.772	1.827	1.521	3.232	Nonane	C_9_H_20_
7.774	1.234	2.957	1.887	7.025	4.116	Decane	C_10_H_22_
9.416		2.112	2.752	1.739	4.322	Undecane	C_11_H_24_
10.939	1.386	2.190	6.097	1.837	4.329	Dodecane	C_12_H_26_
12.372	1.697	2.439	5.958	2.077	4.226	Tridecane	C_13_H_28_
13.706	1.384	3.881	4.449	2.068	6.727	Tetradecane	C_14_H_30_
14.881	1.242	2.587	4.252	1.980	10.935	Pentadecane	C_15_H_32_
16.168	1.169	2.936	2.716	1.682	8.981	Hexadecane	C_16_H_34_
17.297	1.511	1.682	3.571	2.419	3.164	Heptadecane	C_17_H_36_
18.372	1.554	2.791	2.462	2.004	1.645	Octadecane	C_18_H_38_
19.387	1.431	2.934	2.109	2.291	1.847	Nonadecane	C_19_H_40_
20.360	2.868	2.652	0.384	0.262		Eicosane	C_20_H_42_
21.283	2.225	1.477	0.119	0.511		Heneicosane	C_21_H_44_
22.172	2.016	1.197	0.302	1.641		Docosane	C_22_H_46_
23.025	2.842	1.494	0.035	1.555		Tricosane	C_23_H_48_
23.845	4.316	0.456	0.173	0.131		Tetracosane	C_24_H_50_
24.635	4.360	0.299		0.546		Pentacosane	C_25_H_52_
25.395	2.461	0.815	0.127	0.432		Hexacosane	C_26_H_54_
26.132	1.249	0.124	0.207	0.410		Heptacosane	C_27_H_56_
26.842	1.031	0.557	0.721	0.401		Octacosane	C_28_H_58_
27.529	1.379					Nonacosane	C_29_H_60_
Branched aliphatic hydrocarbon compounds							
		0.034				Butane, 2-methyl-	C_5_H_12_
1.792	0.556	0.564	0.507	0.558		Pentane, 2-methyl-	C_6_H_14_
1.855		0.064	0.030	1.037		Pentane, 3-methyl-	C_6_H_14_
2.535	0.055	0.059	0.068	0.061		Hexane, 3-methyl-	C_7_H_16_
2.807	0.892	1.568	1.383	1.249	1.302	Heptane	C_7_H_16_
3.729	1.508		1.456	1.317	0.845	Heptane, 4-methyl-	C_8_H_18_
4.632	0.303		0.498	0.397	0.584	Hexane, 2,3,5-trimethyl-	C_9_H_20_
6.121		0.511				1-Ethyl-4-methylcyclohexane	C_9_H_18_
7.916	0.813	1.589	0.565	0.526	1.268	Nonane, 2,6-dimethyl-	C_11_H_24_
7.986	0.891	0.589	0.738	0.666		Decane, 2-methyl-	C_11_H_24_
11.446	0.204					Dodecane, 4,6-dimethyl-	C_14_H_30_
16.072	1.871	2.845	2.436	1.716	2.847	Cetene	C_16_H_32_
17.200	1.832	2.776	1.881	2.521	1.227	1-Heptadecene	C_17_H_34_
17.204	1.543				1.729	1-Nonadecene	C_19_H_38_
18.027			2.035			Heptadecane, 3-methyl-	C_18_H_38_
Olefin hydrocarbon compounds							
1.629	0.206		0.169	0.271		2-Methyl-1-butene	C_5_H_10_
1.689	0.057	0.178	0.055	0.106		1,4-Pentadiene	C_5_H_8_
1.897	1.593	1.952	2.317	2.692	3.244	1-Pentene, 2-methyl-	C_6_H_12_
1.998	0.698	0.894	0.820	0.769		2-Butene, 2,3-dimethyl-	C_6_H_12_
2.044			0.148			2-Hexene	C_6_H_12_
2.087	0.073	0.136	0.083	0.083		2-Pentene, 3-methyl-	C_6_H_12_
2.203	0.276	0.244	0.151	0.168		2,4-Hexadiene, (E,E)-	C_6_H_10_
2.236	0.258	0.308	1.104	1.132		Cyclopentene, 1-methyl-	C_6_H_10_
2.280	0.350	0.358	0.284	0.257		1-Pentene, 2,4-dimethyl-	C_7_H_14_
2.674	0.163					1-Hexene, 2-methyl-	C_7_H_14_
2.706		1.898	1.618	1.594	1.531	1-Heptene	C_7_H_14_
2.854		0.774	0.762	0.597		1,4-Hexadiene, 2-methyl-	C_7_H_12_
2.857	0.819					1,4-Hexadiene, 5-methyl-	C_7_H_12_
2.890		0.195	0.166	0.147		(Z)-2-Heptene	C_7_H_14_
2.946	0.065	0.096	0.075	0.070		1,4-Hexadiene, 5-methyl-	C_7_H_12_
3.361	0.252		0.337	0.309		2,4-Hexadiene, 2-methyl-	C_7_H_12_
4.124		2.258	2.103	1.935	0.384	1-Octene	C_8_H_16_
4.207		0.328	0.264	0.253		1,4-Pentadiene, 2,3,3-trimethyl-	C_8_H_14_
4.366		0.197	0.167	0.140		2-Octene, (Z)-	C_8_H_16_
4.410	0.678	0.572	0.518	0.439		2,2-Dimethyl-3-heptene trans	C_9_H_18_
4.874	1.615		1.654	6.759		2,3-Dimethyl-2-heptene	C_9_H_18_
5.007	4.779	3.742	1.864	3.391	4.331	2,4-Dimethyl-1-heptene	C_9_H_18_
5.139	0.278					1,3-Hexadiene, 3-ethyl-2-methyl-, (Z)-	C_9_H_16_
5.491	0.520					1,3-Heptadiene, 2,3-dimethyl-	C_9_H_16_
5.650	0.228					6,6-Dimethylhepta-2,4-diene	C_9_H_16_
5.873	1.540	2.562	1.371	2.178	1.689	1-Nonene	C_9_H_18_
5.909			0.333	0.311		2,4,6-Trimethyl-3-heptene	C_10_H_20_
6.261	0.234					1,6-Octadiene, 2,5-dimethyl-, (E)-	C_10_H_18_
7.509	0.318					5-Decene, (E)-	C_10_H_20_
7.628	2.084				2.328	1-Decene	C_10_H_20_
7.677	0.409					2-Decene, 7-methyl-, (Z)-	C_11_H_22_
7.837	0.248					cis-4-Decene	C_10_H_20_
7.850		0.350		0.313		2-Decene, (Z)-	C_10_H_20_
7.853			0.341			4-Decene	C_10_H_20_
8.490	0.116					1-Decene, 5-methyl-	C_11_H_22_
8.776	0.569		0.637	0.199		2-Decene, 2,4-dimethyl-	C_12_H_24_
8.832	0.528		0.680	0.537		3-Decene, 2,2-dimethyl-, (E)-	C_12_H_24_
9.087	1.439					5-Ethyl-1-nonene	C_11_H_22_
9.157	1.433					1-Decene, 2,4-dimethyl-	C_12_H_24_
9.283		3.140	2.087	1.462	5.189	1-Undecene	C_11_H_22_
9.476		0.280	0.447	0.242		2-Undecene, (Z)-	C_11_H_22_
9.480	0.144					5-Undecene	C_11_H_22_
10.816	1.795	3.223	2.736	2.371	5.865	1-Dodecene	C_12_H_24_
10.992	0.288	0.353	0.351	0.323		2-Dodecene, (Z)-	C_12_H_24_
12.140				0.538		6-Tridecene, (Z)-	C_13_H_26_
12.143		0.751	0.584			4-Nonene, 5-butyl-	C_13_H_26_
12.253	1.765	3.255	6.491	4.577	5.443	1-Tridecene	C_13_H_26_
13.490		0.818				5-Tetradecene, (Z)-	C_14_H_28_
13.587	1.755	4.259	3.039	2.642	2.415	1-Tetradecene	C_14_H_28_
13.749				0.313		7-Tetradecene	C_14_H_28_
13.756		1.389	0.489			2-Tetradecene, (E)-	C_14_H_28_
13.875				0.183		3-Tetradecene, (E)-	C_14_H_28_
13.879	1.796	2.720	0.229	2.416	2.753	1-Pentadecene	C_15_H_30_
18.276	1.615		1.365	1.887		1-Octadecene	C_18_H_36_
19.298	1.456	2.015	0.958	1.891		1-Nonadecene	C_19_H_38_
20.277	1.304		0.294			1-Eicosene	C_20_H_40_
23.808	4.268			0.333		1-Tetracosene	C_24_H_48_
Cycloalkane hydrocarboon compounds							
1.755	0.120	0.222	0.145	0.215		Cyclopentene	C_5_H_8_
2.144	0.248	0.466	0.316	0.355		Cyclopentane, methyl-	C_6_H_12_
3.544	0.173	0.258	0.237	0.204		Cyclobutane, (1-methylethylidene)-	C_7_H_12_
4.499		0.159		0.136		Cyclopropane, (2,2-dimethylpropylidene)-	C_8_H_14_
4.662	0.321	0.428	0.284	0.295		1-Methyl-2-methylenecyclohexane	C_8_H_14_
4.788	1.493	0.756	0.798	0.753		Cyclohexane, 1,2,4-trimethyl-	C_9_H_18_
4.821		0.345	0.316	0.255	0.431	Cyclohexane, ethyl-	C_8_H_16_
5.222	1.946	1.514	0.245	1.448		Cyclohexane, 1,3,5-trimethyl-	C_9_H_18_
5.912	0.462	0.337				Cyclopentane, 1,2,3,4,5-pentamethyl	C_10_H_20_
6.118				0.423		Cyclohexane, 1,2,3-trimethyl-	C_9_H_18_
6.845		0.388				Cyclohexane, (1-methylethylidene)-	C_9_H_16_
8.968	0.151					1-Isopropyl-1,4,5-trimethylcyclohexane	C_12_H_24_
9.605				0.163		Cyclopentane, 1,2-dipropyl-	C_11_H_22_
10.242	0.326					Cyclohexane, (2,2-dimethylcyclopentyl)-	C_13_H_24_
10.378	0.201					1-Isopropyl-1,4,5-trimethylcyclohexane	C_12_H_24_
12.047	1.454		0.657			Cyclopentadecane	C_15_H_30_
Cycloalkene hydrocarbon compound							
2.601	0.195	0.253	0.201	0.198		Cyclohexene	C_6_H_10_
2.983	0.147		0.272	0.242		Cyclopentene, 1,5-dimethyl-	C_7_H_12_
3.056		0.308	0.189	0.206		Cyclopentane, 1-methyl-2-methylene-	C_7_H_12_
3.096	0.493	0.861	0.683	0.660		Cyclohexane, methyl-	C_7_H_14_
3.245	0.206	0.358	0.312	0.280		Cyclopentane, ethyl-	C_7_H_14_
3.321	0.270	0.296	0.252	0.253		Cyclohexene, 3-methyl-	C_7_H_12_
3.573			0.715	0.649		1-Ethylcyclopentene	C_7_H_12_
3.779	0.408	0.696	0.569	0.517	0.432	Cyclohexene, 1-methyl-	C_7_H_12_
3.932	0.180	0.220	0.193	0.151		3-Methylenecyclohexene	C_7_H_10_
5.070		0.150	0.134			1-Propylcyclopentene	C_8_H_14_
5.123		0.253	0.181	0.185		1-Methyl-2-methylenecyclohexane	C_8_H_14_
6.317	0.246					Cyclopentene, 1,4-dimethyl-5-(1-methylethyl)-	C_10_H_18_
6.841	0.264		0.392	0.285	0.422	Cyclopentene, 1-butyl-	C_9_H_16_
Aromatic hydrocarbon compounds							
5.312		0.106	0.151	0.485	0.368	Ethylbenzene	C_8_H_10_
7.087		0.489	0.715	0.882		Benzene, 1-ethyl-4-methyl-	C_9_H_12_
7.203		0.241				Benzene, 1-ethyl-3-methyl-	C_9_H_12_
7.400	0.122				0.761	Benzene, 1-ethyl-2-methyl-	C_9_H_12_
7.402	0.217		0.293			Mesitylene	C_9_H_12_

**Table 9:** Elemental analyses and physio-chemical properties.

Elemental Analyses	Non Catalyst	AC	Fe/DM	Fe/DM-AC
C	80.43	82.38	82.48	82.65
H	19.57	17.62	17.52	17.35
H/C (mol/mol)	2.92	2.57	2.55	2.52
Kinematic viscosity (mm^2^/s)	36.14	6.37	6.69	4.31
HHV (MJ/kg)	41.27	42.84	44.73	44.92

**Table 10:** Independent parameters and levels used for CCD.

Parameter	Experiment Factor	Level				
		−α	−1	0	1	+α
Temperature (°C)	A	426.36	440	460	480	493.64
N_2_ flow rate (mL/min)	B	32.96	50	75	100	117.04
catalyst loading (%)	C	8.30	10	12.50	15	16.70

## Data Availability

The data will be made available upon request.

## Supplementary Materials

The following supporting information can be downloaded at: https://www.mdpi.com/article/10.3390/molecules30132884/s1, Figure S1. TGA/DTG of polyethylene; Figure S2. TGA/DTG of polypropylene; Figure S3. FT-IR spectra of the PPW (outer side); Figure S4. FT-IR spectra of PPW (inner side); Table S1. The product distribution analyses according to ASTM D86; Table S2. The synergy of Fe/DM-AC mass/molar ratio reveals the product distribution; Table S3. Testing the model using the sum of squares for pyrolysis oil yield responses; Table S4. Testing the lack of fit for pyrolysis oil yield responses; Table S5. Predicted model responses for pyrolysis oil yield responses; Table S6. Testing the model using the sum of squares for kerosene-like fraction responses; Table S7. Testing the lack of fit for kerosene-like fraction responses; Table S8. A prediction model response for kerosene-like fraction responses; Table S9. Optimization of pyrolysis oil and kerosene-like fraction via CCD and RSM.
