# Supplementary material for: Optimization of Kerosene-like Fuels Produced via Catalytic Pyrolysis of Packaging Plastic Waste via Central Composite Design and Response Surface Methodology: Performance of Iron-Doped Dolomite and Activated Carbon
Source: Molecules. 2025 Jul 7;30(13):2884. doi: 10.3390/molecules30132884 (PMC12250723; doi:10.3390/molecules30132884)
Supplement: Supplementary file 1 [file molecules-30-02884-s001.zip › molecules-3649157-supplementary.pdf]

Supporting Information

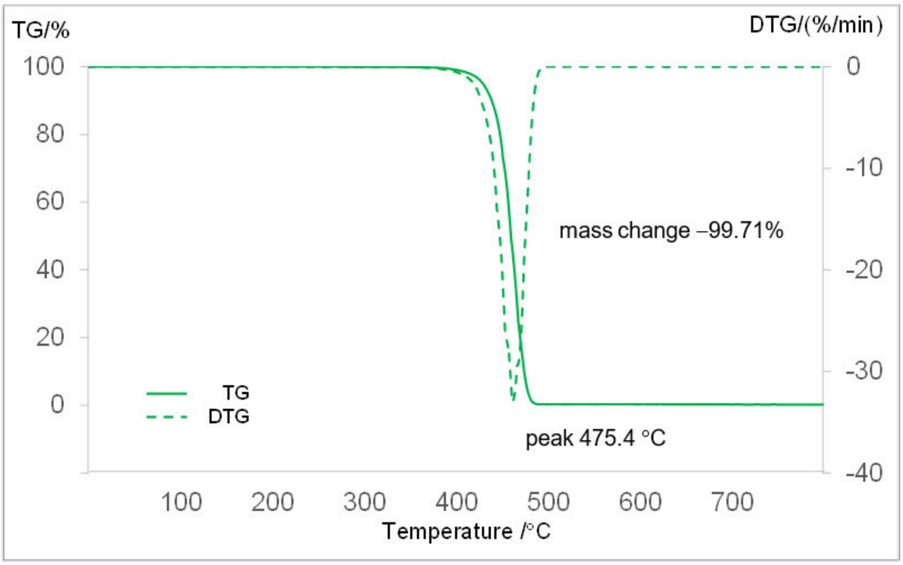

Figure S1. TGA/DTG of polyethylene

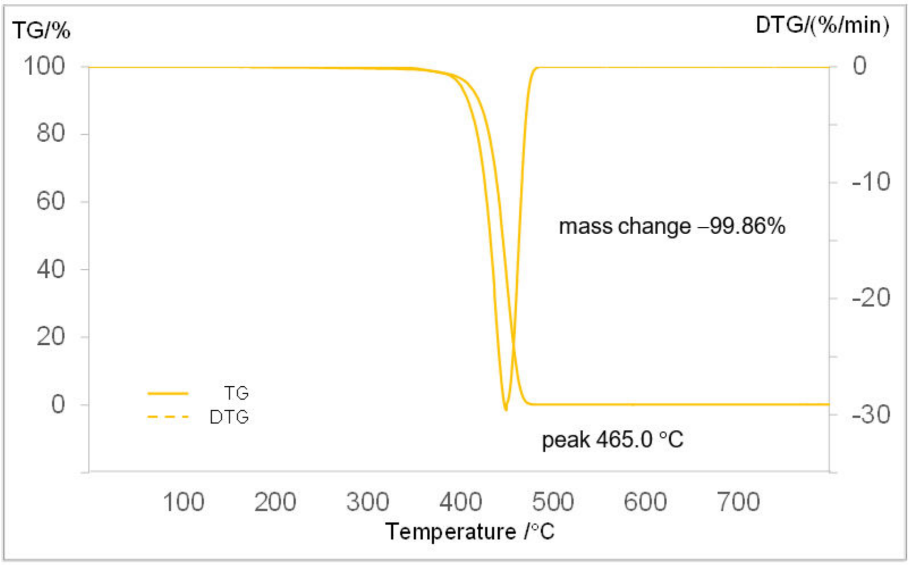

Figure S2. TGA/DTG of polypropylene

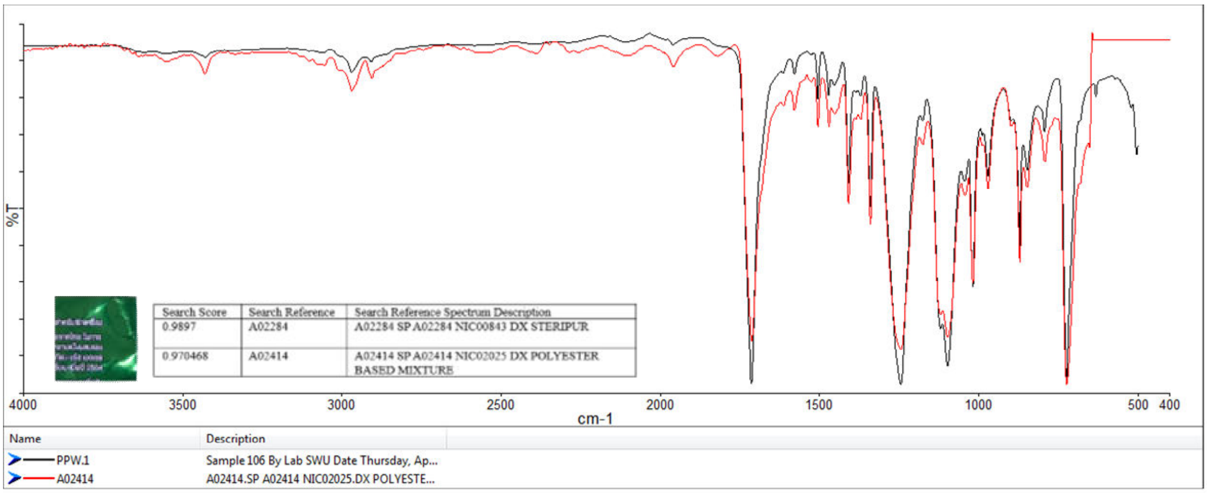

Figure S3. FT-IR spectra of the PPW (outer side)

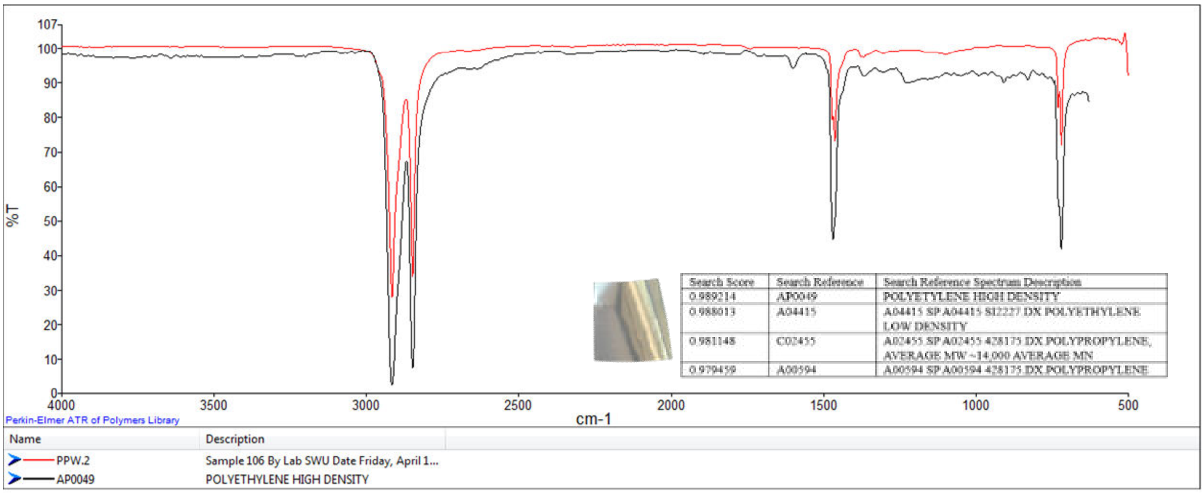

Figure S4. FT-IR spectra of PPW (inner side)

Table S1. The product distribution analyses according to ASTM D86

Product distribution (wt.%)						
The effect of catalyst active site using AC, DM, and Fe-modification dolomite catalyst						
Catalytic activity	solid	LR	Diesel	kerosene	naphtha	gas
non catalyst	9.5 ± 0.20	40.5 ± 0.25	14.0 ± 0.16	3.3 ± 0.21	1.5 ± 0.13	31.2 ± 0.27
AC	4.1 ± 0.08	25.0 ± 0.27	21.1 ± 0.21	11.6 ± 0.23	24.1 ± 0.19	14.1 ± 0.14
DM	6.9 ± 0.12	28.0 ± 0.44	30.8 ± 0.11	10.8 ± 0.14	8.8 ± 0.06	14.8 ± 0.20
5Fe/DM	6.8 ± 0.07	24.7 ± 0.28	21.4 ± 0.24	21.4 ± 0.13	10.6 ± 0.25	16.1 ± 0.14
10Fe/DM	7.3 ± 0.05	22.7 ± 0.15	18.0 ± 0.22	18.3 ± 0.22	13.6 ± 0.37	20.1 ± 0.23
The effect of temperature						
Temperature (°C)	Solid	LR	diesel	kerosene	naphtha	gas
420	6.1 ± 0.04	31.9 ± 0.35	25.1 ± 0.07	15.6 ± 0.11	8.5 ± 0.29	13.0 ± 0.21
440	5.5 ± 0.07	24.7 ± 0.25	20.3 ± 0.34	21.0 ± 0.49	12.6 ± 0.23	15.9 ± 0.13
460	5.9 ± 0.09	22.8 ± 0.52	16.2 ± 0.23	18.2 ± 0.24	15.3 ± 0.15	21.6 ± 0.12
480	7.8 ± 0.17	20.4 ± 0.33	15.8 ± 0.34	16.9 ± 0.16	10.9 ± 0.13	28.3 ± 0.11
500	8.8 ± 0.19	22.9 ± 0.23	16.6 ± 0.17	10.9 ± 0.34	10.4 ± 0.24	30.4 ± 0.12
The effect of N_2 flow rate						
N_2 flow rate (mL/min)						
25	6.9 ± 0.12	20.2 ± 0.32	17.8 ± 0.18	16.9 ± 0.19	9.6 ± 0.22	28.7 ± 0.12
50	5.5 ± 0.07	24.7 ± 0.25	20.3 ± 0.34	21.0 ± 0.49	12.6 ± 0.23	15.9 ± 0.13
75	7.4 ± 0.06	25.8 ± 0.27	26.0 ± 0.12	14.7 ± 0.13	10.5 ± 0.18	15.7 ± 0.10
100	9.9 ± 0.10	28.9 ± 0.49	28.0 ± 0.23	11.0 ± 0.43	6.9 ± 0.14	15.3 ± 0.10
125	10.3 ± 0.07	34.4 ± 0.47	29.3 ± 0.18	8.1 ± 0.12	2.2 ± 0.09	15.7 ± 0.13
The effect of catalyst loading to feedstock						
Catalyst loading (wt.%)	Solid	LR	Diesel	kerosene	naphtha	Gas
non catalyst	8.0 ± 0.03	44.4 ± 0.17	14.6 ± 0.11	1.7 ± 0.17	1.2 ± 0.14	30.2 ± 0.10
5%	5.5 ± 0.07	24.7 ± 0.25	20.3 ± 0.34	21.0 ± 0.12	12.6 ± 0.23	15.9 ± 0.13
10%	5.3 ± 0.16	20.9 ± 0.22	18.0 ± 0.27	21.0 ± 0.28	18.1 ± 0.11	16.7 ± 0.21
15%	5.8 ± 0.11	15.3 ± 0.35	13.2 ± 0.11	20.8 ± 0.13	22.2 ± 0.20	22.8 ± 0.15
20%	7.1 ± 0.08	12.1 ± 0.18	11.4 ± 0.31	19.8 ± 0.12	20.9 ± 0.17	28.7 ± 0.17

The effect of 5Fe/DM blended to AC						
Fe/DM:AC ratio	Solid	LR	diesel	kerosene	naphtha	gas
Fe/DM	6.8 ± 0.08	22.7 ± 0.35	20.4 ± 0.16	21.4 ± 0.27	11.6 ± 0.11	16.1 ± 0.12
0.8:0.2	6.8 ± 0.07	24.4 ± 0.26	20.4 ± 0.37	21.3 ± 0.19	11.2 ± 0.16	16.0 ± 0.13
0.6:0.4	6.8 ± 0.11	23.1 ± 0.19	20.8 ± 0.21	22.3 ± 0.22	13.4 ± 0.25	16.0 ± 0.19
0.5:0.5	5.3 ± 0.16	20.9 ± 0.22	18.0 ± 0.27	21.0 ± 0.28	18.1 ± 0.11	16.7 ± 0.21
0.4:0.6	5.4 ± 0.14	21.2 ± 0.21	18.2 ± 0.13	21.3 ± 0.18	18.2 ± 0.12	15.7 ± 0.11
0.2:0.8	5.4 ± 0.14	25.0 ± 0.14	19.3 ± 0.22	15.7 ± 0.24	20.2 ± 0.18	14.5 ± 0.12
AC	4.1 ± 0.07	25.0 ± 0.15	21.1 ± 0.28	11.6 ± 0.16	24.1 ± 0.21	14.1 ± 0.11

Table S2. The synergy of Fe/DM-AC mass molar ratio reveals the product distribution

	The synergistic effect (%)					
Fe/DM:AC ratio	solid	LR	diesel	kerosene	naphtha	gas
0.8:0.2	8.63	0.58	-0.68	11.11	–20.57	1.91
0.6:0.4	18.88	0.74	0.58	21.85	–33.73	4.58
0.5:0.5	–2.75	–14.17	–13.25	27.27	1.40	10.60
0.4:0.6	4.25	–13.40	–17.39	43.69	–4.71	5.37
0.2:0.8	16.38	1.05	–7.92	15.78	–6.48	0.00

Table S3. Testing the model using the sum of squares for pyrolysis oil yield responses

Source	Sum of Squares	df	Mean Square	F value	p value Prob > F	
Mean vs Total	97995.8	1	97995.8			
Linear vs Mean	782.483653	3	260.8279	10.349045	0.0005	Suggested
2FI vs Linear	72.5218375	3	24.17395	0.950212	0.4450	
Quadratic vs 2FI	169.705159	3	56.56839	3.5130755	0.0570	Suggested
Cubic vs Quadratic	120.887397	4	30.22185	4.5180281	0.0504	Aliased
Residual	40.1350088	6	6.689168			
Total	99181.5331	20	4959.077			

Table S4. Testing the lack of fit for pyrolysis oil yield responses

Source	Sum of Squares	df	Mean Square	F value	p value Prob > F	
Linear	402.854669	11	36.62315	463.89738	< 0.0001	Suggested
2FI	330.332831	8	41.2916	523.03163	< 0.0001	
Quadratic	160.627673	5	32.12553	406.92705	< 0.0001	Suggested
Cubic	39.7402755	1	39.74028	503.3813	< 0.0001	Aliased
Pure Error	0.39473333	5	0.078947			
Linear	402.854669	11	36.62315	463.89738	< 0.0001	Suggested
2FI	330.332831	8	41.2916	523.03163	< 0.0001	

Table S5. Predicted model responses for pyrolysis oil yield responses

Source	Std. Dev.	R-square	Adjusted	Std. Dev.	R-square	
Linear	5.02026769	0.659916	0.5961497	0.38772	726.0006	Suggested
2Fl	5.04386577	0.721078	0.5923441	0.2288908	914.3297	
Quadratic	4.01275972	0.8642	0.7419802	0.0279299	1218.85	Suggested
Cubic	2.58634262	0.966152	0.8928138	6.3884238	8760.698	Aliased

Table S6. Testing the model using the sum of squares for kerosene-like fraction responses

Source	Sum of Squares	df	Mean Square	F value	p value Prob > F	
Mean vs Total	8651.136	1	8651.136			
Linear vs Mean	38.340	3	12.780	11.990	0.0002	
2FI vs Linear	10.289	3	3.430	6.590	0.0060	Suggested
Quadratic vs 2FI	1.072	3	0.357	0.628	0.6135	
Cubic vs Quadratic	5.313	4	1.328	20.925	0.0011	Aliased
Residual	0.381	6	0.063			
Total	8706.531	20	435.327			

Table S7. Testing the lack of fit for kerosene-like fraction responses

Source	Sum of Squares	df	Mean Square	F value	p value Prob > F	
Linear	16.826	11	1.530	33.504	0.0006	
2FI	6.537	8	0.817	17.898	0.0028	Suggested
Quadratic	5.465	5	1.093	23.941	0.0017	Suggested
Cubic	0.153	1	0.153	3.341	0.1271	Aliased
Pure Error	0.228	5	0.046			

Table S8. A prediction model response for kerosene-like fraction responses

Source	Std. Dev.	R-square	Adjusted	Std. Dev.	R-square	
Linear	1.032	0.634	0.435	31.299	1.032	Suggested
2Fl	0.721	0.821	0.539	25.557	0.721	
Quadratic	0.755	0.805	0.243	41.942	0.755	Suggested
Cubic	0.252	0.978	0.387	33.958	0.252	Aliased

Table S9. Optimization of pyrolysis oil and kerosene like fraction via CCD and RSM

run	Temperature (°C)	N_2 flow rate (mL/min)	catalyst loading (wt.%)	pyrolysis oil (wt.%)	kerosene-like (wt.%)	desirability
1	440	50	10	79.91	23.51	0.86
2	440	50.27	10	79.96	23.5	0.86
3	440	50	10.02	79.87	23.51	0.86
4	440	50	10.05	79.83	23.51	0.86
5	440	51.59	10	80.19	23.42	0.86
6	440.02	51.27	10	80.12	23.44	0.86
7	440	51.83	10	80.23	23.41	0.86
8	440	50.06	10.09	79.78	23.51	0.86
9	440	52.45	10	80.33	23.38	0.86
10	440	52.64	10	80.36	23.37	0.86
11	440	50	10.13	79.72	23.52	0.86
12	440.2	50.01	10	79.8	23.48	0.86
13	440	53.99	10	80.56	23.29	0.86
14	440	54.74	10	80.67	23.25	0.86
15	440.19	52.86	10	80.29	23.33	0.86
16	440	50.01	10.39	79.33	23.53	0.86
17	440.59	50	10	79.58	23.43	0.85
18	440	57.95	10	81.05	23.07	0.85
19	440	52.31	10.56	79.51	23.41	0.85
20	440	50	10.67	78.93	23.54	0.85
21	440	58.69	10	81.13	23.03	0.85
22	440	58.89	10	81.14	23.02	0.85
23	440	50	10.84	78.7	23.55	0.85
24	440	60.12	10	81.25	22.95	0.85

run	Temperature (°C)	N_2 flow rate (mL/min)	catalyst loading (wt.%)	pyrolysis oil (wt.%)	kerosene-like (wt.%)	desirability
25	440	60.53	10	81.28	22.93	0.84
26	440	60.95	10	81.31	22.9	0.84
27	440	50.33	11.17	78.33	23.55	0.84
28	440	50	11.23	78.17	23.57	0.84
29	440	50	11.9	77.35	23.6	0.83
30	440	50	12	77.24	23.6	0.82
31	440	52.11	12.08	77.59	23.5	0.82
32	440	57.99	11.88	78.78	23.19	0.82
33	440	67.18	10	81.56	22.56	0.82
34	440	68.05	10	81.56	22.51	0.82
35	440	54.27	12.63	77.44	23.42	0.82
36	440	57.85	12.58	78.09	23.25	0.82
37	440	51.49	12.64	76.88	23.56	0.82
38	440	50.01	12.66	76.53	23.63	0.81
39	440	55.11	12.88	77.37	23.4	0.81
40	440	59.72	12.74	78.21	23.17	0.81
41	440	52.68	13.06	76.73	23.52	0.81
42	440	57.7	13.12	77.61	23.29	0.81
43	440	58.42	13.15	77.7	23.26	0.81
44	440	54.56	13.23	76.97	23.44	0.81
45	440	59.87	13.09	77.95	23.18	0.81
46	440	54.59	13.36	76.87	23.45	0.81
47	440	62.82	12.71	78.59	23.01	0.81
48	440	54.97	13.38	76.92	23.43	0.81
49	440	56.85	13.46	77.21	23.35	0.81
50	440	57.61	13.47	77.33	23.31	0.81
51	440	57.02	13.58	77.15	23.35	0.81
52	440	55.19	13.55	76.84	23.43	0.81
53	440.14	63.13	12.94	78.38	23	0.8
54	440	57.67	14.1	76.92	23.35	0.8
55	440	57.81	14.18	76.89	23.35	0.8
56	440	59.19	14.45	76.98	23.31	0.8
57	440	59.89	14.44	77.1	23.28	0.8
58	440	61.06	14.46	77.26	23.23	0.8
59	440	59.85	14.56	77.03	23.29	0.8
60	440	58.98	14.57	76.88	23.33	0.8
61	440	61.31	14.52	77.27	23.22	0.8
62	440	59.17	14.66	76.87	23.32	0.8
63	440	59.67	14.67	76.95	23.3	0.8
64	440	59.98	14.68	77	23.29	0.8
65	440	61.01	14.68	77.16	23.25	0.8
66	440	61.89	14.55	77.34	23.2	0.8
67	440	59.79	14.75	76.94	23.3	0.8
68	440	59.91	14.77	76.95	23.3	0.8
69	440	60.14	14.91	76.92	23.3	0.8
70	440	61.19	14.89	77.1	23.25	0.8
71	440	60.48	14.99	76.95	23.29	0.8

run	Temperature (°C)	N_2 flow rate (mL/min)	catalyst loading (wt.%)	pyrolysis oil (wt.%)	kerosene-like (wt.%)	desirability
72	440.02	60.9	14.99	77.01	23.27	0.8
73	440	62.11	15	77.19	23.22	0.8
74	440	64.03	15	77.45	23.14	0.8
75	440.02	68.75	15	77.91	22.94	0.79
76	440	74.03	10	81.36	22.18	0.79
77	440	70.83	15	78.05	22.86	0.79
78	440	74.91	15	78.19	22.68	0.78
79	440	77.94	15	78.17	22.56	0.77
80	440	79.66	15	78.12	22.48	0.77
81	453.35	100	15	72.24	21.42	0.6
